# Supplementary material for: Metapipeline-DNA: A comprehensive germline and somatic genomics Nextflow pipeline
Source: Cell Rep Methods. 2026 Mar 17;6(3):101340. doi: 10.1016/j.crmeth.2026.101340 (PMC13030954; doi:10.1016/j.crmeth.2026.101340)
Supplement: Document S1. Figures S1 and S2 and Tables S1 and S2 [file mmc1.pdf]

Cell Reports Methods, Volume *6*

Supplemental information

Metapipeline-DNA: A comprehensive germline

and somatic genomics Nextflow pipeline

Yash Patel, Chenghao Zhu, Takafumi N. Yamaguchi, Nicholas K. Wang, Nicholas Wiltsie, Nicole Zeltser, Alfredo E. Gonzalez, Helena K. Winata, Yu Pan, Mohammed Faizal Eeman Mootor, Timothy Sanders, Sorel T. Fitz-Gibbon, Cyriac Kandoth, Julie Livingstone, Lydia Y. Liu, Benjamin Carlin, Aaron Holmes, Jieun Oh, John Sahrmann, Shu Tao, Stefan Eng, Rupert Hugh-White, Kiarod Pashminehazar, Arpi Beshlikyan, Madison Jordan, Selina Wu, Mao Tian, Jaron Arbet, Beth Neilsen, Roni Haas, Yuan Zhe Bugh, Gina Kim, Joseph Salmingo, Wenshu Zhang, Aakarsh Anand, Edward Hwang, Anna Neiman-Golden, Philippa Steinberg, Wenyan Zhao, Prateek Anand, Raag Agrawal, Brandon L. Tsai, and Paul C. Boutros

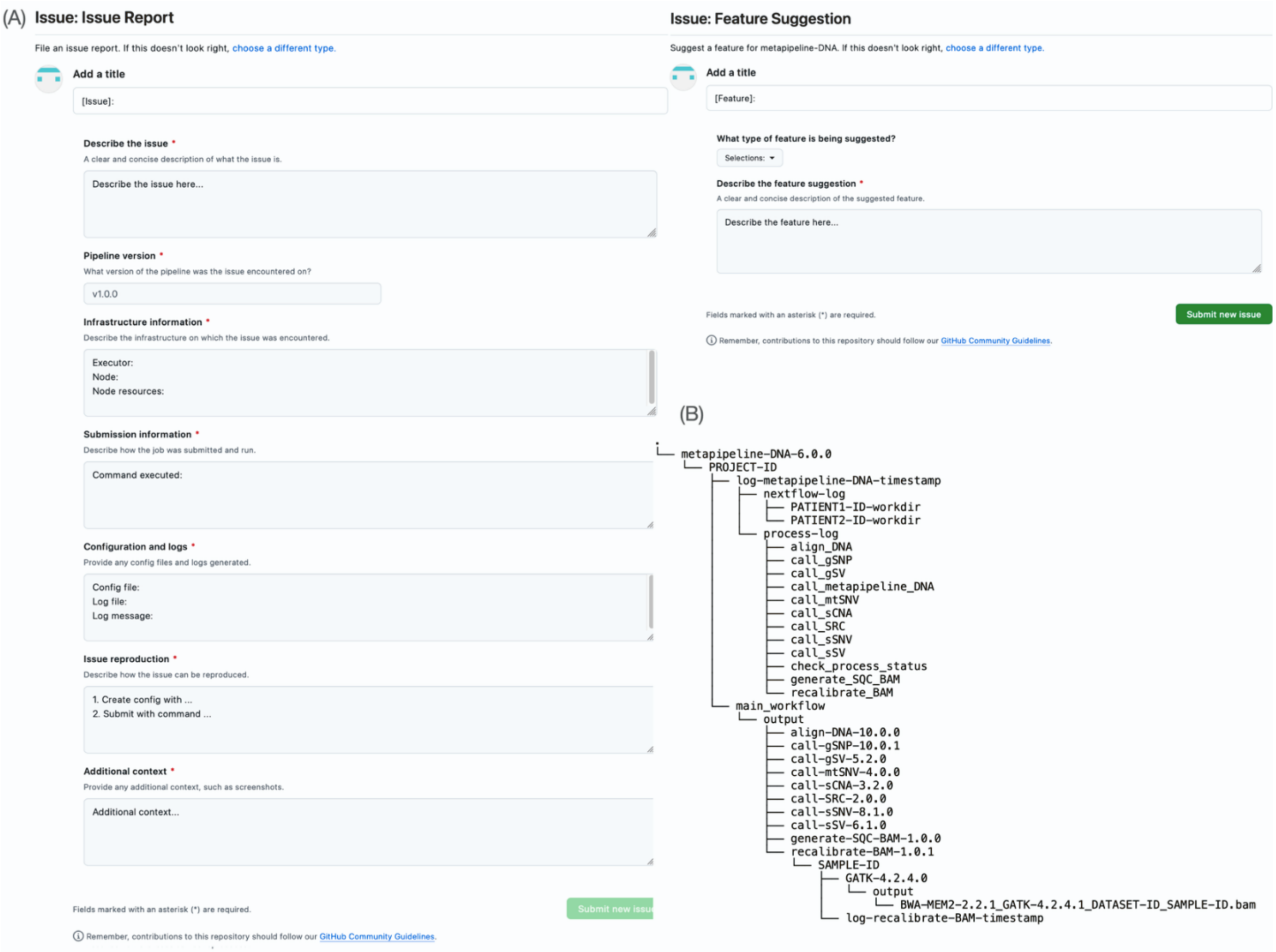

Supplementary Figure 1: **Reporting templates and output directory structure, related to Figure 1. (A)** Issue forms for submitting bug reports and feature suggestions, with structured input options to describe the bug/feature. **(B)** Outputs are organized under each pipeline with a sample/patient/project identifier followed by a directory for logs and a directory for each main tool used in the pipeline. Metapipeline-DNA outputs follow the same structure with individual pipelines' outputs organized recursively in metapipeline-DNA's output.

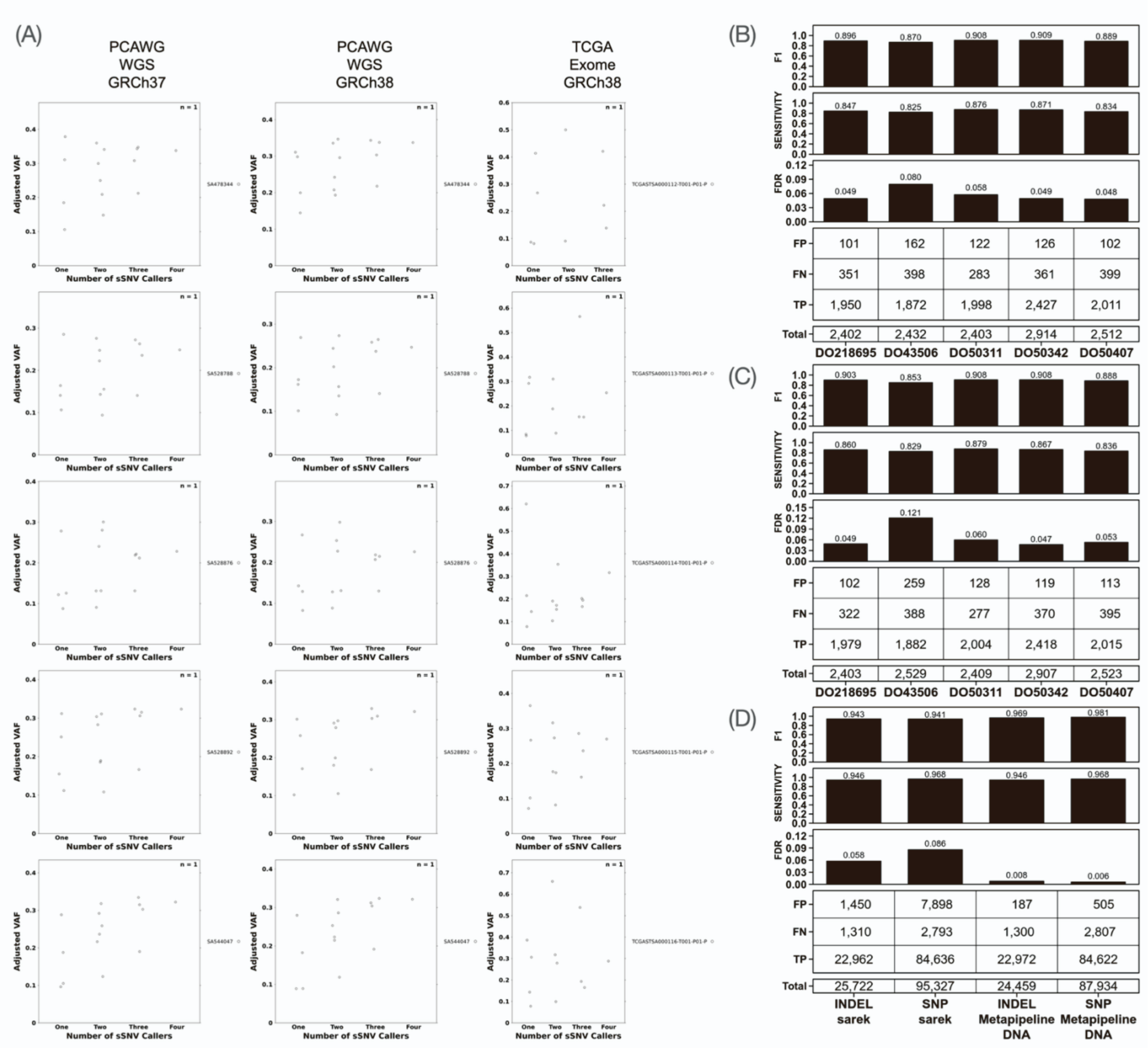

Supplementary Figure 2: VAF plots for all samples with variant calling comparison between sarek and metapipeline-DNA, related to Figures 1 and 2. (A) Variant allele frequencies based on consensus between callers for all samples processed. (**B)** TP, FN and FP variant calls comparing sarek Mutect2 SNV calls from the PCAWG-5 samples against a set of validation variant calls made from targeted deep-sequencing of the same samples. Numbers represent the number of variant calls. **(C)** TP, FN and FP variant calls comparing metapipeline-DNA Mutect2 SNV calls from the PCAWG-5 samples against a set of validation variant calls made from targeted deep-sequencing of the same samples. Numbers represent the number of variant calls. (**D)** TP, FN and FP variant calls comparing germline SNP and INDEL calls from sarek and metapipeline-DNA against the GIAB HG002 truth set. Numbers represent the number of variant calls.

Pipeline	Input Data	Sample Modes	Output Artefacts	Algorithms
Convert-BAM2FASTQ (https://github.com/uclahs-cds/pipeline-convert-BAM2FASTQ)	BAM/CRAM – Aligned reads in BAM or CRAM format	Single sample	FASTQ – Raw reads extracted per readgroup	SAMtools v1.15.1
Align-DNA (https://github.com/uclahs-cds/pipeline-align-DNA)	FASTQ – Paired raw reads with information about sequencing such as readgroup, library, sequencing center	Single sample	BAM – Aligned reads in BAM format	BWA-MEM2 v2.2.1<br>HISAT2 v2.2.1
Calculate-targeted-coverage (https://github.com/uclahs-cds/pipeline-calculate-targeted-coverage)	BAM – Aligned reads in BAM format<br>Target region BED – Genomic sites targeted for sequencing	Single sample	Expanded regions<br>Per-base depth in target regions and dbSNP sites<br>Hybrid-selection metrics	SAMtools v1.16.1<br>BEDtools v2.29.2
Recalibrate-BAM (https://github.com/uclahs-cds/pipeline-recalibrate-BAM)	BAM – Aligned reads in BAM format<br>*Target regions – Genomic sites targeted for sequencing/analysis*	Single sample<br>Normal-tumour paired samples<br>Multi-normal and/or multi-tumour samples	INDEL realigned and base-quality score recalibrated BAM	GATK v3.7.0, v4.2.4.1
Generate-SQC-BAM (https://github.com/uclahs-cds/pipeline-generate-SQC-BAM)	BAM – Aligned reads in BAM format (typically including INDEL realignment and BQSR)	Single sample	BAM statistics – Statistics related to alignment, reads, quality, duplication<br>Coverage metrics – Statistics and plots of coverage across the genome	SAMtools v1.18<br>Picard v3.1.0<br>Qualimap v2.3
Call-gSNP (https://github.com/uclahs-cds/pipeline-call-gSNP)	BAM - Aligned reads in BAM format (typically including INDEL realignment and BQSR)<br>*Target regions - Genomic sites targeted for sequencing/analysis*	Single sample<br>Normal-tumour paired samples<br>Multi-normal and/or multi-tumour samples	Per-sample GVCF – Genomic VCF generated per sample<br>Germline SNP VCF – Recalibrated and filtered germline SNP calls for set of given samples	GATK v4.2.4.1<br>DeepVariant v1.9.0
Call-mtSNV (https://github.com/uclahs-cds/pipeline-call-mtSNV)	BAM/CRAM – Aligned reads in BAM or CRAM format (typically including INDEL realignment and BQSR)	Single sample<br>Norma-tumour paired samples	Mitochondrial SNV VCF	MToolBox v1.2.1-b52269e<br>mitoCaller v1.0.0
Call-gSV (https://github.com/uclahs-cds/pipeline-call-gSV)	BAM – Aligned reads in BAM format (typically including INDEL realignment and BQSR); single normal BAM	Single sample	Germline SV BCF – Variant calls made by DELLY in BCF format<br>Germline SV BCF – Variant calls made by Manta in VCF format	DELLY v1.2.6<br>Manta v1.6.0
Call-sSV https://github.com/uclahs-cds/pipeline-call-sSV)	BAM – Aligned reads in BAM format	Normal-tumour paired samples	Somatic SV BCF – Variant calls made	DELLY v1.2.6<br>Manta v1.6.0<br>SVision v1.4

	(typically including INDEL realignment and BQSR)		by DELLY in BCF format<br>Somatic SV VCF – Variant calls made by Manta in VCF format	
Call-sSNV<br>https://github.com/uclahs-cds/pipeline-call-sSNV)	BAM – Aligned reads in BAM format (typically including INDEL realignment and BQSR)<br>*Somatic SNV calls – Variant calls provided in VCF format to run the consensus call workflow*<br>*Panel of normal – PON used with Mutect2 to improve variant calls*	Single tumour sample<br>Normal-tumour paired samples<br>Multiple tumour samples	Somatic SNV VCFs – Variant calls made by each of the algorithms, VCFs separated per algorithm and per variant type (SNV, MNV, INDEL) when applicable	Mutect2 v4.5.0.0<br>Strelka2 v2.9.10<br>SomaticSniper v1.0.5.0<br>MuSE v2.0.4<br>DeepSomatic v1.9.0<br>BCFtools-Intersect v1.17
Call-sCNA<br>(https://github.com/uclahs-cds/pipeline-call-sCNA)	BAM – Aligned reads in BAM format (typically including INDEL realignment and BQSR)	Normal-tumour paired samples	Somatic CNA TSV – Aberrations called by Battenberg in TSV format<br>Somatic CNA VCF – Aberrations called by FACETS in VCF format	Battenberg v2.2.9<br>FACETS v0.16.0
Call-SRC<br>(https://github.com/uclahs-cds/pipeline-call-SRC)	SNV calls – Generated by any of the algorithms from call-sSNV<br>CNA calls – Generated by any of the algorithms from call-sCNA and HATCHet	Single tumour sample<br>Multiple tumour samples	SNV clustering – Result of clustering of SNVs by clustering algorithms<br>Reconstructed phylogeny	PyClone v0.13.1<br>PyClone-VI v0.1.2<br>PhyloWGS v2205be1<br>DPClust v75f5d7e<br>FastClone v1.0.9<br>CliP v1.3<br>CONIPHER v2.2.0
StableLift<br>(https://github.com/uclahs-cds/pipeline-StableLift)	Variant calls – Generated by any of the following algorithms: HaplotypeCaller, Mutect2, Strelka2, SomaticSniper, MuSE2, DELLY2	Single sample	Lifted variant calls – Variant calls lifted over into the target reference genome<br>Variant stability score – Predicted score of variant stability across reference genome builds	BCFtools v1.20<br>StableLift v1.0.0
Call-GeneticAncestry<br>(https://github.com/uclahs-cds/pipeline-call-GeneticAncestry)	Germline variant calls – Generated by any germline variant caller	Cohort of samples	Predicted genetic ancestry	ADMIXTURE v1.3.0<br>PLINK2 v2.00a4.5lm
Annotate-VCF<br>(https://github.com/uclahs-cds/pipeline-annotate-VCF)	Variant calls – Generated by any caller in VCF format	Single sample	Annotated variant calls – Variant calls annotated with the selected databases	SnpEff v5.1d<br>Funcotator v4.2.4.1<br>VEP v101.0
Calculate-mtDNA-CopyNumber<br>(https://github.com/uclahs-	Genomic coverage – Coverage information per-contig to be used	Single sample	Calculated mitochondrial DNA copy number	

cds/pipeline-calculate-mtDNA-CopyNumber)	in calculating mitochondrial DNA copy number			

Supplementary Table 1: Detailed pipeline inputs, outputs, and tools, related to Table 1. Detailed description of inputs, outputs, run modes, and tools encompassed in metapipeline-DNA. Inputs that are *italicized* are optional and inputs separated by "/" represent a list of choices from which one must be chosen.

Pipeline	PCAWG WGS GRCh37 (wall-clock time in hours)	PCAWG WGS GRCh37 (Peak RAM in GB)	PCAWG WGS GRCh38 (wall-clock time in hours)	PCAWG WGS GRCh38 (Peak RAM in GB)	TCGA WXS GRCh38 (wall-clock time in hours)	TCGA WXS GRCh38 (Peak RAM in GB)
Align-DNA (normal)	4.92 ± 1.68	38.12 ± 1.36	5.56 ± 2.04	50.82 ± 1.73	0.40 ± 0.12	24.82 ± 0.69
Align-DNA (tumour)	7.06 ± 0.69	38.48 ± 1.98	8.20 ± 0.86	51.42 ± 5.07	0.39 ± 0.09	24.96 ± 0.62
Recalibrate-BAM	31.01 ± 4.64	21.20 ± 0.44	31.31 ± 3.08	20.62 ± 0.06	2.10 ± 0.44	2.84 ± 0.07
Generate-SQC-BAM	3.80 ± 2.17	0.41 ± 0.66	4.96 ± 0.67	1.53 ± 0.08	0.34 ± 0.07	0.92 ± 0.009
Call-gSNP	13.14 ± 1.16	5.36 ± 0.07	7.27 ± 3.41	5.42 ± 0.06	0.41 ± 0.11	5.06 ± 0.07
Call-mtSNV	3.43 ± 0.17	10.56 ± 4.06	3.23 ± 0.42	10.58 ± 4.01	0.096 ± 0.024	6.46 ± 0.11
Call-sSNV	12.04 ± 2.92	48.54 ± 2.30	10.52 ± 1.83	43.7 ± 6.98	0.43 ± 0.07	29.32 ± 3.82
Call-sSV	12.85 ± 6.60	9.47 ± 6.55	18.89 ± 3.01	13.14 ± 0.29	0.44 ± 0.11	8.60 ± 0.91
Call-gSV	7.33 ± 2.09	7.24 ± 1.90	8.05 ± 2.83	7.48 ± 1.80	0.34 ± 0.08	2.10 ± 0.09
Call-sCNA	3.43 ± 0.17	46.0 ± 5.67	3.23 ± 0.42	45.14 ± 5.89	2.72 ± 0.05	19.26 ± 0.07
Call-SRC	2.39 ± 0.96	0.41 ± 0.09	2.58 ± 1.01	0.41 ± 0.09	0.014 ± 0.002	0.27 ± 0.04
**TOTAL**	**83.36 ± 12.99**	**-**	**81.76 ± 14.23**	**-**	**6.05 ± 0.80**	**-**

Supplementary Table 2: Runtime and peak physical memory usage of pipelines per sample with 95% confidence intervals, related to Table 1. The total runtime is less than the sum of the individual pipelines' runtimes due to parallelization of variant calling pipelines.
