# Supplementary material for: Metapipeline-DNA: A comprehensive germline and somatic genomics Nextflow pipeline
Source: Cell Rep Methods. 2026 Mar 17;6(3):101340. doi: 10.1016/j.crmeth.2026.101340 (PMC13030954; doi:10.1016/j.crmeth.2026.101340)
Supplement: Document S2. Article plus supplemental information [file mmc2.pdf]

Cell Reports
Methods

Report

Metapipeline-DNA: A comprehensive germline and somatic genomics Nextflow pipeline

Graphical abstract

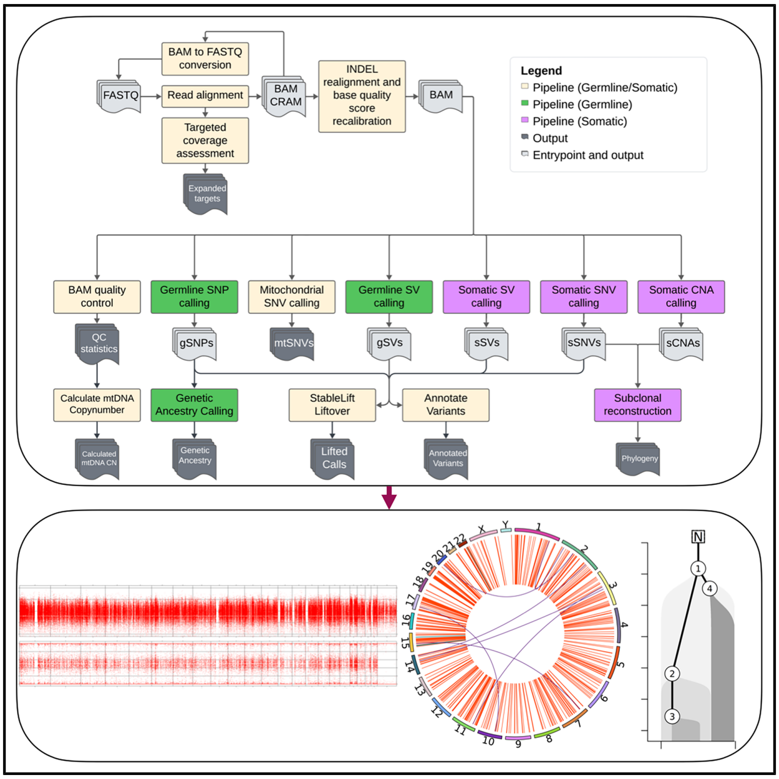

Authors

Yash Patel, Chenghao Zhu,
Takafumi N. Yamaguchi, ...,
Raag Agrawal, Brandon L. Tsai,
Paul C. Boutros

Correspondence

pboutros@sbpdiscovery.org

In brief

Patel et al. develop an automated, extensible, and cloud-compatible DNA sequencing analysis pipeline for DNA sequencing data to transform raw sequencing reads into genetic characteristics and evolutionary features. They demonstrate and validate the pipeline using whole-genome and targeted sequencing data from normal and tumor samples.

Highlights

- Metapipeline-DNA is a computational pipeline to analyze DNA sequencing data
- Metapipeline-DNA identifies genetic and evolutionary features from DNA
- Metapipeline-DNA is automated, highly customizable, and compute-agnostic
- Metapipeline-DNA is optimized for disk usage and allocation of available resources

Patel et al., 2026, Cell Reports Methods 6, 101340
March 23, 2026 © 2026 The Author(s). Published by Elsevier Inc.
https://doi.org/10.1016/j.crmeth.2026.101340

Cell Reports Methods

CellPress
OPEN ACCESS

Report

Metapipeline-DNA: A comprehensive germline and somatic genomics Nextflow pipeline

Yash Patel,[1,2,3,6,8] Chenghao Zhu,[1,2,8] Takafumi N. Yamaguchi,[1,2,3,6,8] Nicholas K. Wang,[1,2] Nicholas Wiltsie,[1,2,3] Nicole Zeltser,[1,2,4] Alfredo E. Gonzalez,[1,2] Helena K. Winata,[1,2] Yu Pan,[1,2,3] Mohammed Faizal Eeman Mootor,[1,2,3] Timothy Sanders,[1,2,3] Sorel T. Fitz-Gibbon,[1,2,3] Cyriac Kandoth,[1,2] Julie Livingstone,[1,2,4] Lydia Y. Liu,[1,2,4] Benjamin Carlin,[1,2,3] Aaron Holmes,[1,2] Jieun Oh,[1,2] John Sahrmann,[1,2] Shu Tao,[1,2,3] Stefan Eng,[1,2] Rupert Hugh-White,[1,2,6] Kiarod Pashminehazar,[1,2] Arpi Beshlikyan,[1,2] Madison Jordan,[1,2] Selina Wu,[1,2] Mao Tian,[1,2,6] Jaron Arbet,[1,2] Beth Neilsen,[1,2] Roni Haas,[1,2] Yuan Zhe Bugh,[1,2] Gina Kim,[1,2] Joseph Salmingo,[1,2] Wenshu Zhang,[1,2] Aakarsh Anand,[1,2] Edward Hwang,[1,2] Anna Neiman-Golden,[1,2] Philippa Steinberg,[1,2] Wenyan Zhao,[1,2,6] Prateek Anand,[1,2] Raag Agrawal,[1,2,4] Brandon L. Tsai,[1,2] and Paul C. Boutros[1,2,3,4,5,6,7,*]
[1]Department of Human Genetics, University of California, Los Angeles, Los Angeles, CA, USA
[2]Jonsson Comprehensive Cancer Center, University of California, Los Angeles, Los Angeles, CA, USA
[3]Institute for Precision Health, University of California, Los Angeles, Los Angeles, CA, USA
[4]Department of Urology, University of California, Los Angeles, Los Angeles, CA, USA
[5]Broad Stem Cell Research Center, University of California, Los Angeles, Los Angeles, CA, USA
[6]Sanford Burnham Prebys Medical Discovery Institute, La Jolla, CA, USA
[7]Lead contact
[8]These authors contributed equally
*Correspondence: pboutros@sbpdiscovery.org
https://doi.org/10.1016/j.crmeth.2026.101340

MOTIVATION Rapid improvements in DNA sequencing technologies have expanded the breadth of genomic features, ranging from nuclear, mitochondrial, and evolutionary variation in germline and somatic contexts, which can be elucidated from sequencing data. In parallel, analytical workflows required to process and identify these features have become increasingly complex, relying on specialized tools and algorithms with varying assumptions and computational requirements. Comprehensive analysis, therefore, requires significant integration effort, limiting scalability, reproducibility, and consistent quality controls. To address this need for a flexible, robust framework that accommodates diverse sequencing methods and feature classes while being highly scalable and adaptable across computational environments, we created metapipeline-DNA to automate genomic analyses.

SUMMARY

The price, quality, and throughput of DNA sequencing continue to improve. Algorithmic innovations have allowed inference of a growing range of features from DNA sequencing data, quantifying nuclear, mitochondrial, and evolutionary aspects of both germline and somatic genomes. To automate analyses of the full range of genomic characteristics, we created an extensible Nextflow metapipeline called metapipeline-DNA. It analyzes targeted and whole-genome sequencing data from raw reads through preprocessing, feature detection by multiple algorithms, quality control, and data-visualization. Each step can be run independently and is supported by robust software engineering including automated failure-recovery, granular testing, and consistent verifications of inputs, outputs, and parameters. Metapipeline-DNA is cloud-compatible and highly configurable, with options to subset and optimize each analysis. Metapipeline-DNA facilitates high-scale, comprehensive analysis of DNA sequencing data, and is open-source under the GPLv2 license.

INTRODUCTION

High-throughput technologies have made biomedical research increasingly data-intensive. DNA sequencing is a key enabling technology, used both in routine clinical care and to support a wide range of research studies.[1] Ongoing improvements in DNA sequencing continue to reduce costs and enable new discoveries, like elucidation of complex structural variants (SVs) and

© 2026 The Author(s). Published by Elsevier Inc.
This is an open access article under the CC BY license (http://creativecommons.org/licenses/by/4.0/).

repetitive genomic regions by long-read sequencing.[2] Modern germline DNA sequencing studies routinely quantify single-nucleotide polymorphisms (SNPs), SVs, telomere length, mitochondrial copy number and variation, copy number, and many other features.[3–5]

DNA sequencing has been especially helpful in characterizing tumors. In many studies both a sample of a cancer tissue and a "reference" normal sample from the same individual are sequenced to better distinguish somatic from germline variation and enable analysis of germline-somatic interactions. Cancers often exhibit widespread genomic rearrangements and clonal variation in mutation burden, specific patterns of somatic mutations associated with carcinogens or other features and a host of features absent or uncommon in germline genomes, like kataegis and chromothripsis.[6] Comprehensive analyses of cancer sequencing can improve diagnosis, prognosis, and management.[7,8]

The growing availability of DNA sequencing has been paralleled by rapid development and adoption of both specific algorithms and workflow software. New discoveries often rely heavily on complex workflows comprising a mixture of established and novel algorithms.[9] These workflows, often termed "pipelines," are implemented in a range of orchestration frameworks including Galaxy,[10] Snakemake,[11] Common Workflow Language (CWL),[12] and Nextflow.[13] Workflows provide a way to automate processes by minimizing manual handling of data flow and facilitating stitching together of different tools to process raw data into refined forms such as lists of variants or quantitation of specific features.

The use of complex workflows has placed a growing emphasis on standardization, extensibility, quality control, and compute infrastructure. Workflow implementations routinely differ across research groups, with many groups creating their own. Many workflows lack key features like unit testing, integration testing, error-handling, fault-tolerance, input-output verification, quality-control, data-visualization, and use of multiple algorithms to create consensus calls.[14] Given the volume of data and the expense of compute, workflows are often bespoke to the high-performance computing environment used by a single group.[15] Portability of workflows to new environments is part of the "model to data" (M2D) paradigm in data sharing and processing.[16] M2D overcomes the cost, time, and privacy risks of data-transfer by bringing models or algorithms to the computing system where data are stored. M2D thus necessitates that models be portable across providers and environments to support workflow usage in conjunction with good data management principles hinging on findability, accessibility, interoperability, and reusability.[17]

To address the need for a robust open-source DNA sequencing analysis pipeline, we created metapipeline-DNA. This Nextflow metapipeline is highly customizable and is capable of processing data from any stage of analysis. It can process DNA sequencing data starting from raw reads through alignment and recalibration, variant calling and even highly integrated analyses likely tumor subclonal reconstruction. Extensive quality control, testing, and data-visualization are built into each individual step and into the full metapipeline. It can work on multiple compute systems and clouds, facilitating analyses at any scale.

RESULTS

Overview

Metapipeline-DNA is a Nextflow metapipeline for the analysis of DNA sequencing data. It can analyze both targeted and whole-genome sequencing with 16 pipelines (Table 1) that collectively transform raw sequencing reads into sets of detected variants and other genetic and evolutionary features (Figure 1A). Most individual pipelines execute multiple algorithms and create consensus calls. For example, subclonal copy number aberration (CNA) detection uses two algorithms (FACETS and Battenberg) and produces visualizations including logR and B-allele frequency (BAF) plots (Figure 1B). Similarly four separate algorithms can be executed for somatic single-nucleotide variant (SNV) detection,[14] automatically generating a consensus set of predictions and variant-associated data-visualizations (Figures 1C and 1D). Each pipeline can be executed independently and can be extensively parameterized to customize the selection and tuning of algorithms.

Several different sample run-modes are available, which we denote with the terminology *n*T-*m*N, where *n* indicates the number of tumor samples and *m* the number of reference samples (Figure 1E). Thus, classic paired tumor-normal analysis is 1T-1N. Metapipeline-DNA fully supports modes like 0T-1N (i.e., germline DNA sequencing), 0T-3N (e.g., family trios), 1T-0N (i.e., unpaired tumour-only sequencing), and arbitrary multiregion tumor sequencing (e.g., 5T-1N). The primary limitation to multisample analyses is compute resource availability—particularly RAM and scratch-disk space. Metapipeline-DNA automatically handles input types for each mode and only executes feasible pipelines, independent of user-selections. For example, in 0T modes, variant detection is restricted to germline variants without users having to provide manual guidance or parameterization.

The default mode of metapipeline-DNA accepts unaligned reads in FASTQ[18] format and executes all pipelines. A range of alternative entry-points are possible, including starting from an unaligned BAM, an aligned BAM,[19] or from CRAM files, with automatic BAM-to-FASTQ conversions as needed. A few pipelines accept alternative entry-points, such as SNV and CNA calls for tumor subclonal reconstruction,[20] germline SNPs for determination of genetic ancestry and germline SNPs or somatic SNVs for variant functional annotation (Figure 1A). Documentation of all dependencies, input and output formats is available on standardized structured GitHub pages: current states at writing are summarized in Table S1.

We engineered metapipeline-DNA to be intrinsically flexible. All dependencies are automatically identified and executed. All run-modes and settings defaults set to the most common behavior across thousands of runs, with easy re-parameterization. For example, when input data are already aligned the default is to use these alignments. Configuration parameters allow the user to control whether reads are converted to FASTQ and re-aligned and whether aligned reads are recalibrated and so forth.

In a similar way, metapipeline-DNA is flexible to the specific genome build used and has been tested extensively with GRCh37, GRCh38, and GRCm39. It can run in two

Table 1. Metapipeline-DNA constituent pipelines

Pipeline	Input formats	Output artifacts	Algorithms	Features
Convert-BAM2FASTQ	BAM/CRAM	FASTQ	SAMtools	automatic detection of and conversion from CRAM to BAM before reversion to FASTQ in the event of CRAM input support for both BAM and CRAM inputs
Align-DNA	FASTQ	BAM	BWA-MEM2 HISAT2	duplicate marking
Calculate-targeted-coverage	BAM target region BED	expanded regions per-base depth in target regions and dbSNP sites hybrid-selection metrics	SAMtools BEDtools	automatic expansion of regions to off-target dbSNP loci with coverage
Recalibrate-BAM	BAM<br>**target regions**	INDEL realigned and base-quality score recalibrated BAM	GATK	support for target regions local INDEL realignment base-quality score recalibration
Generate-SQC-BAM	BAM	BAM statistics<br>coverage metrics	SAMtools Picard Qualimap	customizable selection of QC<br>coverage reporting and visualization
Call-gSNP	BAM<br>**target regions**	per-sample GVCF germline SNP VCF	GATK DeepVariant	variant quality score recalibration<br>ambiguous variant filtration
Call-mtSNV	BAM/CRAM	mitochondrial SNV VCF	MToolBox mitoCaller	mitochondrial read extraction support for BAM and CRAM heteroplasmy calling
Call-gSV	BAM	germline SV VCF germline SV BCF	DELLY Manta	germline CNV calling variant call QC
Call-sSV	BAM	somatic SV VCF somatic SV BCF	DELLY Manta SVision	germline SV filtration
Call-sSNV	BAM<br>**somatic SNV calls**<br>**panel of normal**	somatic SNV VCFs	Mutect2 Strelka2 SomaticSniper MuSE DeepSomatic BCFtools-Intersect	support for panel of normals tumor-only mode<br>multi-tumor mode consensus callset and visualization<br>variant allele frequency distribution by callset
Call-sCNA	BAM	somatic CNA VCF or TSV	Battenberg FACETS	standardized visualization<br>Option for customizing Battenberg refit suggestions
Call-SRC	SNV calls<br>CNA calls	SNV clustering<br>reconstructed phylogeny	PyClone PyClone-VI PhyloWGS DPClust FastClone CliP CONIPHER	Customizable combinations of clustering algorithm and phylogeny algorithm<br>standardized clustering and phylogeny formats
StableLift	variant calls (gSNP, sSNV, sSV, gSV)	lifted variant calls in target reference genome	StableLift	customizable direction of liftover<br>customizable selection of model for stability prediction
Call-Genetic Ancestry	germline variant calls	called genetic ancestry	ADMIXTURE<br>PLINK2	support for VCF and PLINK inputs
Annotate-VCF	variant calls	Annotated variant calls	SnpEff<br>Funcotator<br>VEP	support for variant normalization<br>customizable selection of annotation databases
Calculate-mtDNA-CopyNumber	sample coverage	mitochondrial copy number calculated based on coverage		support for extensible range of coverage sources

Pipelines encompassed within metapipeline-DNA and their inputs, outputs, algorithms, and key features. Inputs that are bolded are optional and inputs separated by "/" represent a list of choices from which one must be chosen.

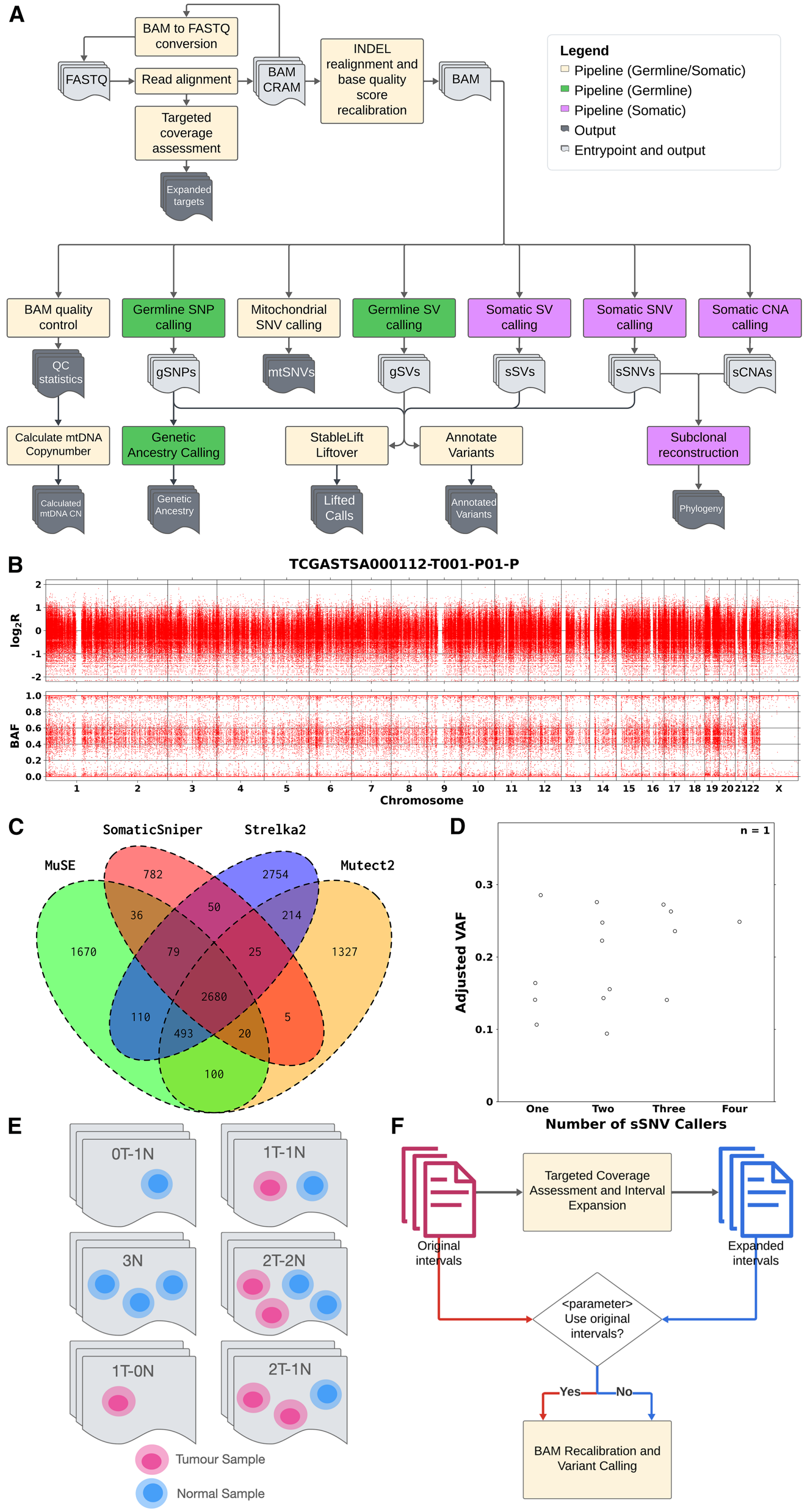

Figure 1. Data flow and visualizations

(A) Data flow through metapipeline-DNA, highlighting the available analyses and modes (somatic, germline, etc.). The different available entry points into metapipeline-DNA are indicated to show flexibility of pipeline selection and input format.

(B) Normalized tumor coverage relative to the matched normal (log_2R) and the B-allele frequency of individual SNPs laid out across the genome to support CNA detection.

(C) Variant calls from 4 SNV callers: MuSE2, SomaticSniper, Strelka2, and Mutect2. Intersection diagram shows consensus sets of variants between the callers.

(D) Variant allele frequencies (VAFs) based on consensus status between callers. VAFs are indicated for all combinations of consensus between one-, two-, three-, and four-variant callers, with each data point representing one combination. The adjusted VAF is calculated as an average of all variants present in the combination.

(E) Input sample combinations supported by metapipeline-DNA. The nT-mN (e.g., 2T-2N) combination indicates any arbitrary numbers of normal and tumor samples. Each combination is automatically detected based on the provided inputs and considered during processing of all pipelines to select appropriate algorithms and processing modalities.

(F) Automatic and customizable interval usage in metapipeline-DNA. Original intervals undergo assessment and expansion at dbSNP sites with high coverage to produce expanded intervals that cover high-coverage off-target regions. The option of using the expanded or the original intervals for downstream BAM recalibration and variant calling is parameterized with both options automated.

modes: WGS mode and targeted-sequencing mode, based on user parameterization. Targeted-sequencing mode supports all subsets of the genome, including exomes and arbitrary panels. Options are available to assess coverage, evaluate covered off-target sites and to automatically use expanded target intervals for downstream processing (Figure 1F).

Data visualization and quality-control

Metapipeline-DNA includes a range of quality control steps and pipelines to assess data at each step, including raw reads, alignments, and variant calls. The pipeline for back-conversion from BAM/CRAM to FASTQ includes built-in checks like SAM flag and alignment statistics assessment and read count comparison before and after conversion to FASTQ (to ensure no loss of reads due to file corruption or parallelization scatter-gather failures, for example). These quality controls produce a variety of data-visualizations and reports. For example, alignment quality is inferred from BAM (or CRAM) files in a range of ways including coverage distributions over the genome (or target region with or without padding; Figures 2A and 2B). Reads are quantified by a range of quality metrics, including total counts, mapping qualities, guanine cytosine (GC) content, insert sizes, read lengths, duplications, and others. Figure 2C shows an example of read number stratified by a range of quality groupings. A range of software are used to generate these metrics, including SAMtools,[19] Picard,[21] and Qualimap.[22] Pileup summaries at common sites are generated and used as a precursor to estimate contamination across samples. Visualization is also built into the SV calling pipelines to produce representations of SVs and their categorization (inversion, insertion, and breakend/translocation) in circos plots (Figure 2D).

In targeted-sequencing mode, additional coverage assessment is performed through per-base read depth calculations at target regions and well-characterized off-target polymorphic sites provided from dbSNP.[23] The workflow also generates an expanded set of targets encompassing the original target regions plus user-defined polymorphic sites (typically dbSNP) enriched in coverage over a user-defined threshold. Metapipeline-DNA provides with configuration to automatically use the expanded targets with BAM recalibration and variant calling pipelines.

Variants are additionally evaluated for stability across reference genomes: StableLift[24] is available as an optional workflow to support liftover of sSVs, gSVs, sSNVs, and gSNPs between GRCh37 and GRCh38. In addition to liftover, the pipeline annotates variants with databases such as dbSNP[23] and applies a model to assign a stability score to each variant to indicate the likelihood of the variant being consistently represented across the two reference genome builds. Variant type-specific assessment is also performed. For example, germline SNP calls undergo filtration using models built from variant quality scores for both SNPs and Indels. Somatic SNVs are assessed based on consensus between callers and associated variant allele frequencies. The consensus approach across callers allows for filtering of SNV calls to reduce the rate of false positives made by a single caller.

Germline SNP calls undergo genetic sex-specific evaluations to reduce the rate of false positives. Variants on chromosomes X and Y in non-pseudo-autosomal regions[25–27] (PARs) are extracted and filtered based on the genetic sex. In XY samples, heterozygous genotype calls are removed, and homozygous genotype calls are converted to hemizygous. In XX samples, all chromosome Y variant calls are removed. This reduces the false positive rate of variant calls made on the sex chromosomes.

Software-engineering and pipeline robustness

We placed a heavy focus on generating reusable and extensible software that could automatically detect and recover from common errors, particularly in the compute environment. This led us to adopt or create a series of development practices and pipeline features aimed at maximizing quality. All software is open-source, available on GitHub (https://github.com/TheBoutrosLab/metapipeline-DNA, with older versions available at https://github.com/uclahs-cds/metapipeline-DNA), with transparent tracking of issues and discussions. Development followed a test-driven approach using NFTest.[14] Metapipeline-DNA has a suite of 95 unit, integration, and regression tests that are run for each new release with testing performed for different stages of execution from end-to-end tests to individual pipeline tests. The tests utilize publicly available simulated sequencing data from the ICGC-TCGA DREAM Somatic Mutation Calling Tumor Heterogeneity (SMC-Het) Challenge[28] subsampled at various sequencing depths to facilitate different tests. Our extensive use of Docker containers allows seamless co-existence of multiple pipeline versions, and the combination of automated testing and containerization facilitates rapid updating with new features or dependency versions. Standardized GitHub issue templates support robust reporting of both bugs and new feature-requests, allowing ideal collaboration (Figure S1A). At writing, development has involved 43 contributors making 1,408 pull-requests and 46 individuals making 1,124 suggestions, feature-requests, and issue-reports across 17 repositories.

Bioinformatics data have high intrinsic variability, and bioinformatics software can be prone to significant numbers of failures—particularly in heterogeneous computing environments. Failure handling is built into metapipeline-DNA to predict and minimize wasted computation. We automated input and parameter validation to catch issues prior to commitment of compute resources.[29] Proactive validation of pipeline parameters is implemented to avoid errors prior to resource commitment. Individual pipelines are modularized and fault-tolerant such that errors or failures in one pipeline stay isolated from and do not terminate other pipelines that are not direct dependencies. Metapipeline-DNA can be easily re-run in cases of failure, triggered starting from prior partial results with a simple parameterization.

All outputs are organized with standardized directory and naming structures (Figure S1B). Filenames have been standardized to provide dataset, organism, and sample information in a consistent way across pipelines. Metapipeline-DNA similarly organizes log-files to ensure saving of and ready access to the metapipeline-DNA logs, individual pipeline-level logs, and compute partition logs. These logs capture execution and resource usage metrics for every process. Robust tooling has been developed around process and pipeline execution to ensure logs are captured for both successful and failing steps

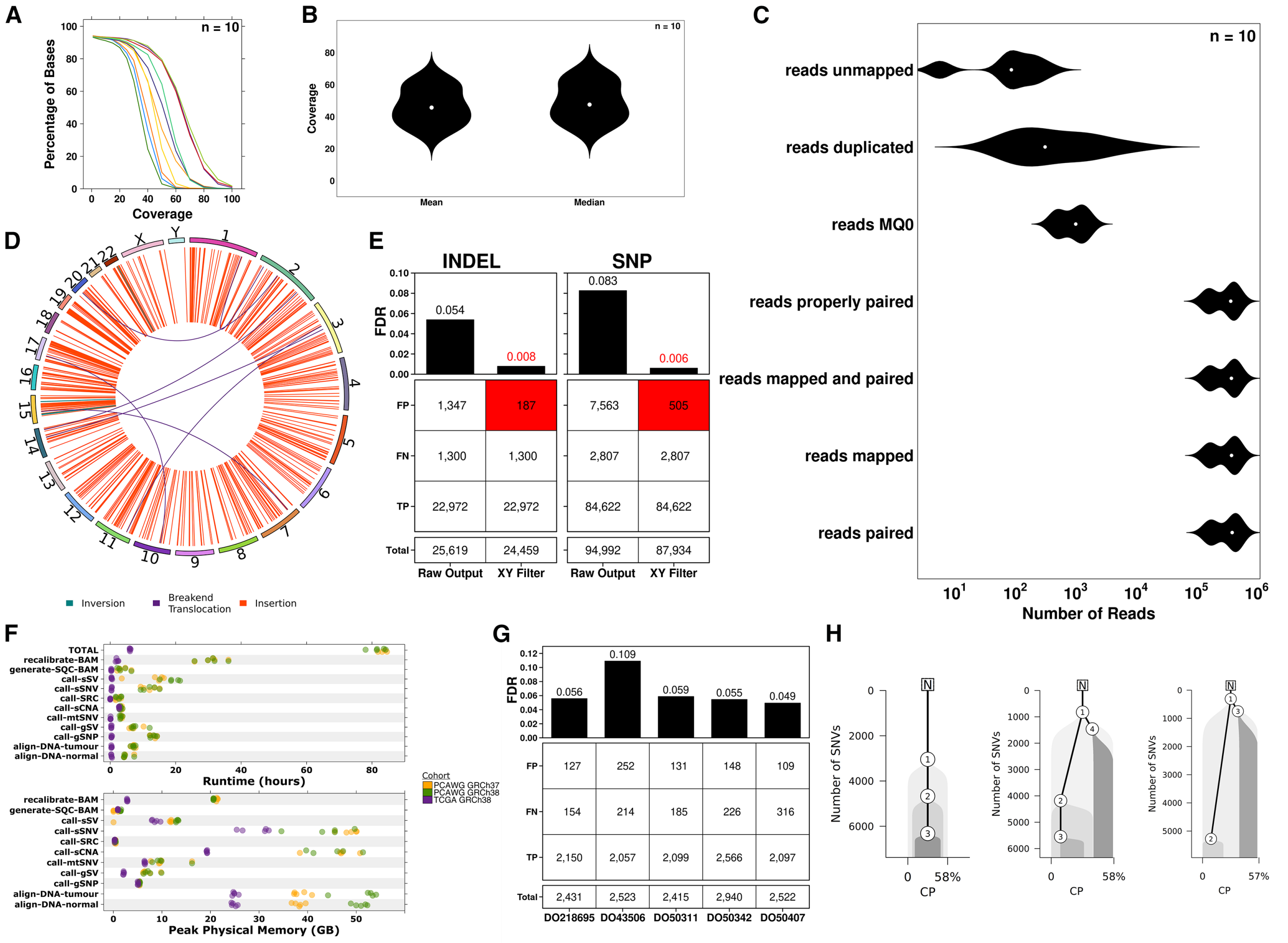

Figure 2. Alignment and coverage metrics

(A) Percent of bases in the genome at each fold of coverage for normal and tumor samples (each line represents one sample) for all five WGS PCAWG patients. Each line represents a different sample, with the percentage of bases calculated using the coverage metrics.

(B) Distribution of mean and median coverage across all samples, highlighting two rough separations arising from the normal samples and tumor samples with the normal samples being in the lower coverage separation.

(C) Distributions of reads across alignment metrics including mapped/unmapped, low mapping quality, duplication, and paired.

(D) Circos plot of somatic SVs categorized into inversions, insertions, and breakend/translocations.

(E) TP, FN, and FP variant calls comparing raw SNP and INDEL calls from HaplotypeCaller- and XY-filtered variant calls against the GIAB HG002 truth set. Numbers represent the number of variant calls with reduction in false discovery rate with XY filtration highlighted in red.

(F) Time and memory usage of pipelines per sample for the three different processing cohorts: PCAWG with GRCh38, PCAWG with GRCh37, and TCGA with GRCh38. Time is measured as wall-clock time for each pipeline and the total time taken by metapipeline-DNA. Memory is measured as the peak RAM usage in GB by any single process by any pipeline.

(G) TP, FN, and FP variant calls comparing consensus call-SNV calls from the PCAWG-5 samples against a set of validation variant calls made from targeted deep-sequencing of the same samples. Numbers represent the number of variant calls.

(H) Reconstructed phylogeny of tumor samples SA478344, SA528788, and SA528876 using a consensus SNV callset comprising variants called by at least two out of four SNV callers (MuSE2, SomaticSniper, Strelka2, and Mutect2) and FACETS CNAs. Nodes represent identified subclones with the evolutionary history depicted over SNV accumulation. Along the x axis is the cellular prevalence (CP), indicating the fraction of all cells comprising each subclone.

to enable debugging and record-keeping. Scripts have been created that automatically "crawl" over a series of pipeline runs to extract and tabulate information about run success, compute resources, and other features.

Compute infrastructure

Metapipeline-DNA includes compute-agnostic customizability of execution and scheduling in distributed workflows. It has been tested and validated on both the Azure and AWS clouds. Execution follows the pattern of a single leading job responsible for submission and monitoring of per-sample or per-patient analysis jobs. Execution is currently performed with the Slurm executor with optional specification of compute partitions.[30] Parameters also exist to control rate of job submission and amount of parallelization/resources usage. Once configured and submitted, metapipeline-DNA automatically handles processing of an

entire cohort with input parsing and job submission without user intervention. Real-time monitoring is available through email notifications sent from a server watching individual step start, end and status. The choice of executor itself is parameterized and can be easily extended to new environments.

Metapipeline-DNA includes optimizations for disk usage, with optional eager intermediate file removal and built in checks to optimize disk usage (e.g., performing I/O operations from high-performance working disks). Resource allocation for individual steps is automatically handled, with pipelines running in parallel as available resources allows. Resource-related robustness is also built into pipelines to detect memory allocation failures from individual tools and automatically retry processes with higher allocations.

Case studies

We assessed the performance of germline SNP XY filtration using the Genome in a Bottle (GIAB) HG002 sample.[31] Variant calls generated by pipeline-call-gSNP were assessed against the GIAB HG002 XY small variant benchmark v1.0 as the truth set.[31] Compared with raw variant calls our XY pipeline decreases false positive INDEL calls from 1,347 to 221 (FDR from 0.054 to 0.009). Similarly, it reduces false-positive SNPs detections from 7,563 to 1,290 (FDR from 0.083 to 0.015). True positive and false negative rates were unchanged, demonstrating improved precision without reduced sensitivity (Figure 2E).

As an additional demonstration and benchmark, 10 normal-tumor pairs were processed through the entirety of metapipeline-DNA. Five pairs were selected from the Pan-Cancer Analysis of Whole Genomes (PCAWG)[32] 63 dataset and another five from The Cancer Genome Atlas (TCGA).[33] The PCAWG-63 samples were sequenced with whole-genome sequencing and derived from multiple cancer types: one from uterine corpus endometrial carcinoma, one from biliary tract carcinoma, and three from esophageal adenocarcinoma. Tumor samples had a median coverage of 63× (range: 45–65×) and normal samples had a median coverage of 38× (range: 34–54×). The TCGA samples were derived from soft tissue sarcoma samples sequenced with exome-targeted sequencing. Both pairs were processed using metapipeline-DNA from alignment to subclonal reconstruction. PCAWG-63 samples were processed with both GRCh38 and GRCh37, with similar runtimes across the two reference builds at an average of 81.2 h (95% CI: ±14.2) for GRCh38 and 83.4 h (95% CI: ±13.0) for GRCh37. Processing was performed in Azure using Fsv2 instances with 72 CPUs and 144 GB of RAM, with a cost of $3.41 per hour of pay-as-you-go computation. With instance reservation, the price reduces to $1.12 per hour. The average cost for processing the PCAWG samples was $280.64 per sample at the pay-as-you-go rate and $92.18 at the reserved instance rate. The average cost for TCGA sample was $20.63 per sample at the pay-as-you-go rate and $6.78 at the reserved instance rate. Across the 10 pairs, memory usage peaked in call-sSNV (average ± 95% CI: 48.5 GB ± 2.3 and 29.3 GB ± 3.8 for PCAWG63 GRCh37 and TCGA GRCh38, respectively) and in align-DNA (average ± 95% CI: 51.4 ± 5.1 for PCAWG63 GRCh38). Runtimes and peak memory usage of metapipeline-DNA are visualized in Figure 2F and summarized in Table S2. Both run-times and memory usage are a function of compute hardware and parameter selection and can be extensively tuned.

We assessed our consensus-based sSNV calling workflow and its consensus callset using the PCAWG samples with targeted deep-sequencing (mean coverage of 653×) validation as a truth set. The true positive rate (TPR) for the samples ranged from 0.87 to 0.93 with false discovery rate (FDR) ranging from 0.05 to 0.11 (Figure 2G). To demonstrate phylogenetic reconstruction, we subsampled 5,000 SNVs and used CNA calls (average 138 per sample; Figure 2H). Variant allele frequencies aggregated over all combinations of consensus calls are shown for all samples in Figure S2A.

Benchmarking analysis

Three pipelines were chosen for comparison against metapipeline-DNA: nf-core/sarek,[34] Sentieon,[35] and DRAGEN.[36] A feature comparison covering accepted inputs, types of analyses available, execution requirements, and infrastructure requirements is detailed in Table 2.

We performed an in-depth benchmarking between nf-core/sarek and metapipeline-DNA using the PCAWG-5 and GIAB HG002 samples. For the PCAWG-5 samples, somatic variants were called using nf-core/sarek and their performance was evaluated through comparison against a validation variant call set derived from targeted deep-sequencing of the same samples. The performance of calls from nf-core/sarek and from metapipeline-DNA is detailed in Figures S2B and S2C, respectively. Both pipelines demonstrate similar performance, with F1 scores ranging from 0.870 to 0.909 for nf-core/sarek calls and 0.853 to 0.908 for metapipeline-DNA calls. For each of the pipeline runs, the memory usage peaked at 83.8 GB for nf-core/sarek and 54.1 GB for metapipeline-DNA. Performance on germline variant calling was also compared using the HG002 sample. Germline variant calls from nf-core/sarek and metapipeline-DNA were compared to the GIAB HG002 XY small variant benchmark as the truth set, with metrics detailed in Figure S2D. Variant calls made using metapipeline-DNA show reduced FDR compared to nf-core/sarek's calls (0.058–0.008 for INDELs and 0.086 to 0.006 for SNPs) and consistently higher F1 scores (0.943–0.969 for INDELs and 0.941 to 0.981 for SNPs).

DISCUSSION

Metapipeline-DNA was designed to facilitate analysis of DNA sequencing data at scale while retaining the configurability and flexibility needed in academic environments. This is a key contrast to field programmable gate array (FPGA) approaches such as DRAGEN, which attain outstanding speed through hardware optimization at the expense of algorithmic flexibility and evolution. As the field of genomics evolves, the ability to quickly integrate and test emerging methods continues to be extremely important, highlighting a limitation of fixed-function hardware solutions.

Metapipeline-DNA fills this key niche of supporting the rapidly expanding volume of sequencing data, supporting a range of existing tools and algorithms and remaining flexible for ongoing expansion. By easing and optimizing the multistep analyses intrinsic to DNA sequencing data, it reduces the barrier to incorporating new methods and analyzing large datasets. Indeed, it is entirely feasible for metapipeline-DNA to leverage and incorporate

Table 2. Comparison of metapipeline-DNA with nf-core/sarek, Sentieon, and DRAGEN

Feature	Metapipeline-DNA	nf-core/sarek	Sentieon	DRAGEN
Input types accepted	FASTQ, BAM, CRAM	FASTQ, BAM, CRAM	FASTQ, BAM, CRAM	FASTQ, BAM, CRAM
Types of variants called	SNVs, Indels, gSNPs, gSVs, sSVs, CNAs, mtSNVs, Genetic Ancestry	SNVs, Indels, gSNPs, gSVs, sSVs, CNAs	SNVs, Indels, gSNPs, gSVs, sSVs, CNAa, repeat expansions, mtSNVs	SNVs, Indels, gSNPs, gSVs, sSVs, CNAs, repeat expansions, mtSNVs
Subclonal reconstruction	yes	no	no	no
Follows GATK best practices?	yes	yes	yes	no
Containerization?	yes, with Docker	yes, with Docker and Singularity	no	no
Infrastructure required	flexible with Nextflow workflow manager, runs on CPU architecture	flexible with Nextflow workflow manager, runs on CPU architecture	flexible with self-contained executables, runs on CPU architecture	proprietary software that requires a combination of CPU and FPGA hardware
Scalability	highly scalable through local machines and cloud-based job schedulers for horizontal scaling	highly scalable through local machines and cloud-based job schedulers for horizontal scaling	individual software is highly optimized for vertical scaling	highly scalable vertically through hardware optimization
Ease of execution	single-command execution of cohort	single-command execution of cohort	command-line tools must be scripted into a workflow for execution	single-command execution of samples
Configurability	selection of tools and parameters through command-line arguments and configuration file	selection of tools and parameters handled through command-line arguments and configuration files	selection of tools and parameters handled through specific calls to software and command-line arguments	selection of tools and parameters through command-line arguments
Documentation	standardized, comprehensive documentation with usage, parameter, input, and output descriptions	standardized, extensive, well-maintained with usage, parameter, input, and output descriptions	comprehensive documentation with usage, parameter, input, and output descriptions	comprehensive documentation and suggestions with usage, parameter, input, and output descriptions

List of genomics pipeline features and their presence and evaluation in each compared pipeline.

FPGA-enabled and graphics processing units (GPU)-accelerated methods directly as part of its modular structure (e.g., for alignment); this is a key area of ongoing development.

Individual pipelines within metapipeline-DNA are modular, creating a plug-and-play architecture that can be adapted to support additional technologies as they become available. Algorithms and workflows for processing long-read data, for example, pose an avenue for expanding the meta-pipeline as such tools mature and long-read datasets become more common. The context of DNA also brings up the possibility of similar meta-pipelines for other biological molecules such as RNA and proteins. Workflows across different biomolecules can share the architecture, automation, and quality-control of metapipeline-DNA in a way that allows improvements to any single pipeline to improve the others. Such workflows are currently under development to provide a similar level of configurability and extensibility for analyses of RNA and protein data.

The large volume of data and size of individual samples in sequencing studies creates a need for optimization of analysis pipelines' data handling. metapipeline-DNA contains several disk usage optimizations to efficiently handle large amounts of data while minimizing I/O operations and cross-file system data movement. The framework connecting analyses automatically identifies necessary outputs from dependent pipelines and makes it available without any redundant copying or duplication. There are additional enhancements that are underway to minimize duplicated data and disk usage of metapipeline-DNA by building plugins to enable moving of files rather than copying when possible and optimizing individual pipelines to avoid shuffling around large output files.

Metapipeline-DNA is a highly customizable DNA sequencing analysis pipeline combining speed and flexibility in a modular framework to enable processing of data at any point from read alignment to tumor subclonal reconstruction. By facilitating the integration of diverse tools and supporting the rapid development of new methodologies, it positions itself as a versatile platform for future enhancements as novel DNA sequencing and analysis methods are developed.

Limitations of the study

The on-going improvements in DNA sequencing technologies and acceleration in data volume necessitate optimization in data and resource handling and integration of novel algorithms. Analysis of sequencing data involves several distinct steps ranging from alignment of raw reads to a reference genome to variant calling to variant annotation and tumor evolution reconstruction. The compute resources required at each step vary, with alignment for example requiring high CPU and disk space usage and variant annotation requiring far fewer resources. To efficiently handle the requirements of different steps while maximizing utilization of available resources, metapipeline-DNA will employ a dynamic allocation selection algorithm trained on thousands of analyses that will leverage sequencing data features, such as read length, number of reads and read quality, to automatically determine and allocate necessary compute resources while avoiding waste.

Although SNV calling has been benchmarked against nf-core/sarek, it does not provide end-to-end accuracy and performance assessment for other analyses, including SVs and CNAs. The tools used for calling these variants have been benchmarked individually previously and are employed in metapipeline-DNA. Combined performance benchmarking for these features will be expanded in future developments.

In addition, sequencing processing often employs cohort-level studies combining information a set of individuals to identify patterns related to disease, evolution, and ancestry. To enhance metapipeline-DNA's ability to handle cohort-level analysis, it will employ a split-apply-combine principle to process individuals in parallel before combining results joint genotyping, population ancestry background prediction and variant frequency across populations.

RESOURCE AVAILABILITY

Lead contact

Further information and requests for resources and code should be directed to and will be fulfilled by the lead contact, Paul C. Boutros (pboutros@sbpdiscovery.org).

Materials availability

This study did not generate new unique reagents.

Data and code availability

- The data underlying this article were accessed from the European Genome Phenome Archive (EGA; https://ega-archive.org/search/pcawg under accession number EGAS00001001692) and the Genomics Data Commons (GDC; https://portal.gdc.cancer.gov/ under dataset ID TCGA). The derived data generated in this research will be shared on reasonable request to the corresponding author.
- Source code has been deposited here: https://doi.org/10.5281/zenodo.15085443. It is also available on GitHub (https://github.com/TheBoutrosLab/metapipeline-DNA with older versions available at https://github.com/uclahs-cds/metapipeline-DNA).
- Any additional information required to reanalyze the data reported in this paper is available from the lead contact upon request.

ACKNOWLEDGMENTS

The authors gratefully acknowledge the ongoing support of all present and past members of the Boutros lab and Andrew Park in providing suggestions, practical use-cases, and support. The authors also acknowledge the Office of Health Informatics and Analytics at UCLA Health IT for their infrastructure support, particularly high-performance compute provisioning, data management, and resource tuning. This study was conducted with the support of the National Institutes of Health through awards (R01CA268380, P30CA016042, R01CA244729, R01CA270108, U2CCA271894, U24CA248265, and U54HG012517), and of the Department of Defense through awards (W81XWH2210247 and W81XWH2210751). N.K.W., H.K.W., J.O., R.A., and C.Z. were supported by the Jonsson Comprehensive Cancer Center Fellowship. A.E.G. was supported by the Howard Hughes Medical Institute Gilliam Fellowship. N.Z. was supported by the National Institutes of Health through awards (T32HG002536 and F31CA281168). L.Y.L. was supported by the Canadian Institutes of Health Research Vanier Fellowship and the Ontario Graduate Scholarship. S.W. was supported by the UCLA Tumor Cell Biology Training Program through the USHHS Ruth L. Kirschstein Institutional National Research Service Award (T32CA009056). B.N. was supported by the National Library of Medicine (T15LM013976) Training Grant and ASCO Young Investigator Award. B.L.T. was supported by the UCLA Cancer Center Support Grant (P30CA016042) and the National Institutes of Health through awards (U2CCA271894, U24CA248265, and R01CA272678). R.H. was supported by EMBO Postdoctoral Fellowship ALTF 1131-2021 and the Prostate Cancer Foundation Young Investigator Award 22YOUN32. R.A. was supported by

awards (T32GM008042 and T32GM152342). Funding for open access charge: (R01CA244729).

AUTHOR CONTRIBUTIONS

Y. Patel, conceptualization, methodology, software, visualization, and writing – original draft; C.Z., conceptualization, methodology, and software; T.N.Y., conceptualization, methodology, and software; N.K.W., validation and software; N.W., software and methodology; N.Z., software and methodology; A.E.G., software and methodology; H.K.W., software and methodology; Y. Pan, software; M.F.E.M., software, methodology, and validation; T.S., software; S.T.F-G., software, methodology, visualization, validation, and data curation; C.K., software and methodology; J.L., software and methodology; L.Y.L., software and methodology; B.C., software; A.H., software; J.O., software; J.S., software; S.T., software; S.E., software; R.H.-W., software and data curation; K.P., software; A.B., software; M.J., software; S.W., software; M.T., software and methodology; J.A., software; B.N., software; R.H., software; Y.Z.B., software; G.K., software; J.S., software; W. Zhang, software; A.A., software; E.H., software; A.N., software; P.S., software; W. Zhao, software; P.A., software; R.A., software; B.L.T., software; P.C.B., conceptualization, project administration, supervision, and writing – review and editing.

DECLARATION OF INTERESTS

P.C.B. sits on the Scientific Advisory Boards of Intersect Diagnostics Inc. and BioSymetrics Inc., and previously sat on that of Sage Bionetworks.

STAR★METHODS

Detailed methods are provided in the online version of this paper and include the following:

- KEY RESOURCES TABLE
- METHOD DETAILS
  - Analysis cohort
  - Alignment and variant calling
  - Benchmarking analysis
- QUANTIFICATION AND STATISTICAL ANALYSIS

SUPPLEMENTAL INFORMATION

Supplemental information can be found online at https://doi.org/10.1016/j.crmeth.2026.101340.

Received: June 9, 2025
Revised: December 3, 2025
Accepted: February 6, 2026
Published: March 17, 2026

REFERENCES

1. Shendure, J., Balasubramanian, S., Church, G.M., Gilbert, W., Rogers, J., Schloss, J.A., and Waterston, R.H. (2017). DNA sequencing at 40: past, present and future. Nature *550*, 345–353.
2. Logsdon, G.A., Vollger, M.R., and Eichler, E.E. (2020). Long-read human genome sequencing and its applications. Nat. Rev. Genet. *21*, 597–614.
3. Ding, J., Sidore, C., Butler, T.J., Wing, M.K., Qian, Y., Meirelles, O., and Busonero, F. (2015). Assessing mitochondrial DNA variation and copy number in lymphocytes of 2,000 Sardinians using tailored sequencing analysis tools. PLoS Genet. *11*, e1005306.
4. Zhang, Y., Liu, X., Wiggins, K.L., Kurniansyah, N., Guo, X., Rodrigue, A.L., Zhao, W., Yanek, L.R., Ratliff, S.M., Pitsillides, A., et al. (2023). Association of Mitochondrial DNA Copy Number With Brain MRI Markers and Cognitive Function: A Meta-analysis of Community-Based Cohorts. Neurology *100*, 1930–1943.
5. Gauthier, J., Vincent, A.T., Charette, S.J., and Derome, N. (2019). A brief history of bioinformatics. Brief. Bioinform. *20*, 1981–1996.
6. Puttick, C., Jones, T.P., Leung, M.M., Galvez-Cancino, F., Liu, J., Varas-Godoy, M., Rowan, A., Pich, O., Martinez-Ruiz, C., Bentham, R., et al. (2024). MHC Hammer reveals genetic and non-genetic HLA disruption in cancer evolution. Nat. Genet. *56*, 2121–2131.
7. Chakravarty, D., and Solit, D.B. (2021). Clinical cancer genomic profiling. Nat. Rev. Genet. *22*, 483–501.
8. Sosinsky, A., Ambrose, J., Cross, W., Turnbull, C., Henderson, S., Jones, L., Hamblin, A., Arumugam, P., Chan, G., Chubb, D., et al. (2024). Insights for precision oncology from the integration of genomic and clinical data of 13,880 tumors from the 100,000 Genomes Cancer Programme. Nat. Med. *30*, 279–289.
9. Cremin, C.J., Dash, S., and Huang, X. (2022). Big data: Historic advances and emerging trends in biomedical research. Current Research in Biotechnology *4*, 138–151.
10. Galaxy Community (2022). The Galaxy platform for accessible, reproducible and collaborative biomedical analyses: 2022 update. Nucleic Acids Res. *50*, W345–W351.
11. Köster, J., and Rahmann, S. (2012). Snakemake – A scalable bioinformatics workflow engine. Bioinformatics *28*, 2520–2522.
12. Crusoe, M.R., Abeln, S., Iosup, A., Amstutz, P., Chilton, J., Tijanić, N., Ménager, H., Soiland-Reyes, S., Gavrilović, B., Goble, C., and Community, T.C. (2022). Methods Included: Standardizing Computational Reuse and Portability with the Common Workflow Language. Commun. ACM *65*, 54–63.
13. Di Tommaso, P., Chatzou, M., Floden, E.W., Barja, P.P., Palumbo, E., and Notredame, C. (2017). Nextflow enables reproducible computational workflows. Nat. Biotechnol. *35*, 316–319.
14. Patel, Y., Zhu, C., Yamaguchi, T.N., Bugh, Y.Z., Tian, M., Holmes, A., Fitz-Gibbon, S.T., and Boutros, P.C. (2024). NFTest: automated testing of Nextflow pipelines. Bioinformatics *40*, btae081.
15. Dash, S., Shakyawar, S.K., Sharma, M., and Kaushik, S. (2019). Big data in healthcare: management, analysis and future prospects. J. Big Data *6*, 54.
16. Ellrott, K., Buchanan, A., Creason, A., Mason, M., Schaffter, T., Hoff, B., Eddy, J., Chilton, J.M., Yu, T., Stuart, J.M., et al. (2019). Reproducible biomedical benchmarking in the cloud: lessons from crowd-sourced data challenges. Genome Biol. *20*, 195. https://doi.org/10.1186/s13059-019-1794-0.
17. Wilkinson, M.D., Dumontier, M., Aalbersberg, I.J., Appleton, G., Axton, M., Baak, A., Blomberg, N., Boiten, J.W., da Silva Santos, L.B., Bourne, P.E., et al. (2016). The FAIR Guiding Principles for scientific data management and stewardship. Sci. Data *3*, 160018.
18. Cock, P.J.A., Fields, C.J., Goto, N., Heuer, M.L., and Rice, P.M. (2010). The Sanger FASTQ file format for sequences with quality scores, and the Solexa/Illumina FASTQ variants. Nucleic Acids Res. *38*, 1767–1771.
19. Li, H., Handsaker, B., Wysoker, A., Fennell, T., Ruan, J., Homer, N., Marth, G., Abecasis, G., and Durbin, R.; 1000 Genome Project Data Processing Subgroup (2009). The Sequence Alignment/Map format and SAMtools. Bioinformatics *25*, 2078–2079.
20. Salcedo, A., Tarabichi, M., Buchanan, A., Espiritu, S.M.G., Zhang, H., Zhu, K., Ou Yang, T.-H., Leshchiner, I., Anastassiou, D., Guan, Y., et al. (2025). Crowd-sourced benchmarking of single-sample tumor subclonal reconstruction. Nat. Biotechnol. *43*, 581–592. https://doi.org/10.1038/s41587-024-02250-y.
21. Broad Institute (2019). Picard Toolkit (Broad Institute, GitHub repository).
22. Okonechnikov, K., Conesa, A., and García-Alcalde, F. (2016). Qualimap 2: advanced multi-sample quality control for high-throughput sequencing data. Bioinformatics *32*, 292–294.
23. Sherry, S.T., Ward, M., and Sirotkin, K. (1999). dbSNP – Database for Single Nucleotide Polymorphisms and Other Classed of Minor Genetic Variation. Genome Res. *9*, 677–679.

24. Wang, N.K., Wiltsie, N., Winata, H.K., Fitz-Gibbon, S., Gonzalez, A.E., and Zeltser, N. (2024). StableLift: Optimized Germline and Somatic Variant Detection Across Genome Builds.. Preprint at bioRxiv. https://doi.org/10.1101/2024.10.31.621401.

25. Flaquer, A., Rappold, G.A., Wienker, T.F., and Fischer, C. (2008). The human pseudoautosomal regions: a review for genetic epidemiologists. Eur. J. Hum. Genet. *16*, 771–779. https://doi.org/10.1038/ejhg.2008.63.

26. Freije, D., Helms, C., Watson, M.S., and Donis-Keller, H. (1992). Identification of a second pseudoautosomal region near the Xq and Yq telomeres. Science *258*, 1784–1787. https://doi.org/10.1126/science.1465614.

27. Veerappa, A.M., Padakannaya, P., and Ramachandra, N.B. (2013). Copy number variation-based polymorphism in a new pseudoautosomal region 3 (PAR3) of a human X-chromosome-transposed region (XTR) in the Y chromosome. Funct. Integr. Genomics *13*, 285–293. https://doi.org/10.1007/s10142-013-0323-6.

28. Salcedo, A., Tarabichi, M., Espiritu, S.M.G., Deshwar, A.G., David, M., Wilson, N.M., Dentro, S., Wintersinger, J.A., Liu, L.Y., Ko, M., et al. (2020). A community effort to create standards for evaluating tumor subclonal reconstruction. Nat. Biotechnol. *38*, 97–107. https://doi.org/10.1038/s41587-019-0364-z.

29. Patel, Y., Beshlikyan, A., Jordan, M., Kim, G., Holmes, A., Yamaguchi, T.N., and Boutros, P.C. (2024). PipeVal: light-weight extensible tool for file validation. Bioinformatics *40*, btae079.

30. Yoo, A.B., Jette, M.A., and Grondona, M. (2003). SLURM: Simple Linux Utility for Resource Management. In Lecture Notes in Computer Science (Springer), p. 2862.

31. Wagner, J., Olson, N.D., McDaniel, J., Harris, L., Pinto, B.J., Jáspez, D., Muñoz-Barrera, A., Rubio-Rodríguez, L.A., Lorenzo-Salazar, J.M., Flores, C., et al. (2025). Small variant benchmark from a complete assembly of X and Y chromosomes. Nat. Commun. *16*, 497. https://doi.org/10.1038/s41467-024-55710-z.

32. Cancer Genome Atlas Research Network Electronic address elizabeth demicco@sinaihealthsystem ca; Cancer Genome Atlas Research Network (2017). Comprehensive and Integrated Genomic Characterization of Adult Soft Tissue Sarcomas. Cell *171*, 950–965.e28.

33. ICGC/TCGA Pan-Cancer Analysis of Whole Genomes Consortium (2020). Pan-cancer analysis of whole genomes. Nature *578*, 82–93.

34. Garcia, M., Juhos, S., Larsson, M., Olason, P.I., Martin, M., Eisfeldt, J., DiLorenzo, S., Sandgren, J., De Ståhl, T.D., Wirta, V., et al. (2018). Sarek: A portable workflow for whole-genome sequencing analysis of germline and somatic variants. Preprint at Bioinformatics. https://doi.org/10.1101/316976.

35. Freed, D., Aldana, R., Weber, J.A., and Edwards, J.S. (2017). The Sentieon Genomics Tools - A fast and accurate solution to variant calling from next-generation sequence data. Preprint at Bioinformatics. https://doi.org/10.1101/115717.

36. Behera, S., Catreux, S., Rossi, M., Truong, S., Huang, Z., and Ruehle, M. (2024). Comprehensive genome analysis and variant detection at scale using DRAGEN. Nat. Biotechnol. *43*, 1177–1191.

37. Landrum, M.J., Lee, J.M., Riley, G.R., Jang, W., Rubinstein, W.S., Church, D.M., and Maglott, D.R. (2014). ClinVar: public archive of relationships among sequence variation and human phenotype. Nucleic Acids Res. *42*, D980–D985. https://doi.org/10.1093/nar/gkt1113.

38. Vasimuddin, M., Misra, S., Li, H., and Aluru, S. (2019). Efficient Architecture-Aware Acceleration of BWA-MEM for Multicore Systems. IEEE Parallel and Distributed Processing Symposium (IEEE).

39. McKenna, A., Hanna, M., Banks, E., Sivachenko, A., Cibulskis, K., Kernytsky, A., Garimella, K., Altshuler, D., Gabriel, S., Daly, M., and DePristo, M.A. (2010). The Genome Analysis Toolkit: a MapReduce framework for analyzing next-generation DNA sequencing data. Genome Res. *20*, 1297–1303.

40. Poplin, R., Chang, P.-C., Alexander, D., Schwartz, S., Colthurst, T., Ku, A., Newburger, D., Dijamco, J., Nguyen, N., Afshar, P.T., et al. (2018). A universal SNP and small-indel variant caller using deep neural networks. Nat. Biotechnol. *36*, 983–987. https://doi.org/10.1038/nbt.4235.

41. Hail Team. Hail 0.2.133-4c60fddb171a. https://hail.is.

42. Illumina. Illumina hap.py. https://github.com/Illumina/hap.py.

43. Alexander, D.H., and Lange, K. (2011). Enhancements to the ADMIXTURE algorithm for individual ancestry estimation. BMC Bioinf. *12*, 246. https://doi.org/10.1186/1471-2105-12-246.

44. Purcell, S., Neale, B., Todd-Brown, K., Thomas, L., Ferreira, M.A.R., Bender, D., Maller, J., Sklar, P., de Bakker, P.I.W., Daly, M.J., and Sham, P.C. (2007). PLINK: a tool set for whole-genome association and population-based linkage analyses. Am. J. Hum. Genet. *81*, 559–575. https://doi.org/10.1086/519795.

45. Rausch, T., Zichner, T., Schlattl, A., Stütz, A.M., Benes, V., and Korbel, J.O. (2012). DELLY: structural variant discovery by integrated paired-end and split-end analysis. Bioinformatics *28*, 333–339.

46. Chen, X., Schulz-Trieglaff, O., Shaw, R., Barnes, B., Schlesinger, F., Källberg, M., Cox, A.J., Kruglyak, S., and Saunders, C.T. (2016). Manta: rapid detection of structural variants and indels for germline and cancer sequencing applications. Bioinformatics *32*, 1220–1222.

47. Park, J., Cook, D.E., Chang, P.-C., Kolesnikov, A., Brambrink, L., Mier, J.C., Gardner, J., McNulty, B., Sacco, S., Keskus, A., et al. (2024). DeepSomatic: Accurate somatic small variant discovery for multiple sequencing technologies. Preprint at Bioinformatics. https://doi.org/10.1101/2024.08.16.608331.

48. Ji, S., Montierth, M.D., and Wang, W. (2022). MuSE: A Novel Approach to Mutation Calling with Sample-Specific Error Modeling. Methods Mol. Biol. *2493*, 21–27.

49. Larson, D.E., Harris, C.C., Chen, K., Koboldt, D.C., Abbott, T.E., Dooling, D.J., Ley, T.J., Mardis, E.R., Wilson, R.K., and Ding, L. (2012). SomaticSniper: identification of somatic point mutations in whole genome sequencing data. Bioinformatics *28*, 311–317.

50. Kim, S., Scheffler, K., Halpern, A.L., Bekritsky, M.A., Noh, E., Källberg, M., Chen, X., Kim, Y., Beyter, D., Krusche, P., and Saunders, C.T. (2018). Strelka2: fast and accurate calling of germline and somatic variants. Nat. Methods *15*, 591–594.

51. Danecek, P., Bonfield, J.K., Liddle, J., Marshall, J., Ohan, V., Pollard, M.O., Whitwham, A., Keane, T., McCarthy, S.A., Davies, R.M., and Li, H. (2021). Twelve years of SAMtools and BCFtools. GigaScience *10*, giab008.

52. P'ng, C., Green, J., Chong, L.C., Waggott, D., Prokopec, S.D., Shamsi, M., Nguyen, F., Mak, D.Y.F., Lam, F., Albuquerque, M.A., et al. (2019). BPG: Seamless, automated and interactive visualization of scientific data. BMC Bioinf. *20*, 42.

53. Chen, H., and Boutros, P.C. (2011). VennDiagram: a package for the generation of highly-customizable Venn and Euler diagrams in R. BMC Bioinf. *12*, 35.

54. Lin, J., Wang, S., Audano, P.A., Meng, D., Flores, J.I., Kosters, W., Yang, X., Jia, P., Marschall, T., Beck, C.R., and Ye, K. (2022). SVision: a deep learning approach to resolve complex structural variants. Nat. Methods *19*, 1230–1233. https://doi.org/10.1038/s41592-022-01609-w.

55. Gu, Z., Gu, L., Eils, R., Schlesner, M., and Brors, B. (2014). circlize Implements and enhances circular visualization in R. Bioinformatics *30*, 2811–2812. https://doi.org/10.1093/bioinformatics/btu393.

56. Shen, R., and Seshan, V.E. (2016). FACETS: allele-specific copy number and clonal heterogeneity analysis tool for high-throughput DNA sequencing. Nucleic Acids Res. *44*, e131.

57. Nik-Zainal, S., Van Loo, P., Wedge, D.C., Alexandrov, L.B., Greenman, C.D., Lau, K.W., Raine, K., Jones, D., Marshall, J., Ramakrishna, M., et al. (2012). The life history of 21 breast cancers. Cell *149*, 994–1007.

58. Gillis, S., and Roth, A. (2020). PyClone-VI: scalable inference of clonal population structures using whole genome data. BMC Bioinf. *21*, 571.

59. Deshwar, A.G., Vembu, S., Yung, C.K., Jang, G.H., Stein, L., and Morris, Q. (2015). PhyloWGS: Reconstructing subclonal composition and evolution from whole-genome sequencing of tumors. Genome Biol. *16*, 35.

60. Xiao, Y., Wang, X., Zhang, H., Ulintz, P.J., Li, H., and Guan, Y. (2020). FastClone is a probabilistic tool for deconvoluting tumor heterogeneity in bulk-sequencing samples. Nat. Commun. *11*, 4469.

61. Cingolani, P., Platts, A., Wang, L.L., Coon, M., Nguyen, T., Wang, L., Land, S.J., Lu, X., and Ruden, D.M. (2012). A program for annotating and predicting the effects of single nucleotide polymorphisms, SnpEff: SNPs in the genome of Drosophila melanogaster strain w1118; iso-2; iso-3. Fly *6*, 80–92. https://doi.org/10.4161/fly.19695.

62. McLaren, W., Gil, L., Hunt, S.E., Riat, H.S., Ritchie, G.R.S., Thormann, A., Flicek, P., and Cunningham, F. (2016). The Ensembl Variant Effect Predictor. Genome Biol. *17*, 122. https://doi.org/10.1186/s13059-016-0974-4.

63. Winata, H.K., Knight, D., Salcedo, A., Wu, S., and Boutros, P.C. (2024). CEV: Visualization of Cancer Evolution. https://github.com/uclahs-cds/public-R-CancerEvolutionVisualization.

64. Purcell, S. PLINK. https://www.cog-genomics.org/plink/.

STAR★METHODS

KEY RESOURCES TABLE

REAGENT or RESOURCE	SOURCE	IDENTIFIER
Deposited data		
PCAWG-63 whole-genome sequencing of 5 tumour-normal pairs	The ICGC/TCGA Pan-Cancer Analysis of Whole Genomes Consortium[33]	EGAS00001001692
TCGA exome sequencing of 5 tumour-normal pairs	The Cancer Genome Atlas Research Network[32]	https://portal.gdc.cancer.gov/
ClinVar	Landrum et al.[37]	https://www.ncbi.nlm.nih.gov/clinvar/
Software and algorithms		
BWA-MEM2	Vasimuddin et al.[38]	v2.2.1
GATK	McKenna et al.[39]	v4.2.4.1
GATK	McKenna et al.[39]	v3.7.0
SAMtools	Li et al.[19]	v1.18
Picard	Broad Institute[21]	v3.1.0
DeepVariant	Poplin et al.[40]	v1.9.0
Hail	Hail Team[41]	v0.2.113
Hap.py	Illumina[42]	v0.3.15
ADMIXTURE	Alexander and Lange[43]	v1.3.0
PLINK	Purcell et al.[44]	2.00a4.5lm
Delly2	Rausch et al.[45]	v1.2.6
Manta	Chen et al.[46]	v1.6.0
mitoCaller	Ding et al.[3]	v1.0.0
DeepSomatic	Park et al.[47]	v1.9.0
MuSE2	Ji et al.[48]	v2.0.4
SomaticSniper	Larson et al.[49]	v1.0.5.0
Strelka2	Kim et al.[50]	v2.9.10
BCFtools	Danecek et al.[51]	v1.17
BPG	P'ng et al.[52]	v7.1.0
VennDiagram	Chen and Boutros[53]	v1.7.4
SVision	Lin et al.[54]	v1.4
circlize	Gu et al.[55]	v0.4.16
CNV_FACETS	Shen and Seshan[56]	v0.16.0
Battenberg	Nik-Zainal et al.[57]	v2.2.9
PyClone-VI	Gillis and Roth[58]	v0.1.2
PhyloWGS	Deshwar et al.[59]	v2205be1
FastClone	Xiao et al.[60]	v1.0.9
StableLift	Wang et al.[24]	v1.0.0
SnpEff	Cingolani et al.[61]	v5.1d
VEP	McLaren et al.[62]	v101.0
CEV	Winata et al.[63]	v2.0.0
PipeVal	Patel et al.[29]	v5.1.0
Nextflow	Di Tommaso et al.[13]	v23.04.2
metapipeline-DNA	This paper	Deposited at https://doi.org/10.5281/zenodo.15085443 and at https://github.com/TheBoutrosLab/metapipeline-DNA; and at https://github.com/uclahs-cds/metapipeline-DNA for older versions

METHOD DETAILS

Analysis cohort

To demonstrate the use of metapipeline-DNA, we chose ten normal-tumour pairs. Five were WGS tumour-normal pairs from PCAWG-63: one from uterine corpus endometrial carcinoma donor DO43506, one from biliary tract carcinoma donor DO218695 and three from esophageal adenocarcinoma donors DO50342, DO50407 and DO50311. Five were exome sequencing pairs of soft tissue sarcoma pairs from TCGA donors TCGA-QQ-A8VD, TCGA-X6-A8C6, TCGA-HS-A5N8, TCGA-DX-A1L2 and TCGA-HB-A2OT.[32,33]

Alignment and variant calling

Sequencing reads were aligned to the GRCh38.p7 reference build including decoy contigs from GATK using BWA-MEM2[38] (v2.2.1) in paired-end mode followed by duplicate marking with MarkDuplicatesSpark using GATK[39] (v4.2.4.1). For the GRCh37 runtime benchmarking, alignment was performed to the GRCh37 reference build including decoy contigs. The resulting alignments were recalibrated through Indel realignment using GATK (v3.7.0) and base-quality score recalibration using GATK (v4.2.4.1). Quality metrics were generated using SAMtools[19] (v1.18) stats and Picard[21] (v3.1.0) CollectWgsMetrics. Germline SNPs were called using DeepVariant[40] (v1.9.0) and HaplotypeCaller from GATK (v4.2.4.1) followed by variant recalibration using GATK (v4.2.4.1). Germline SNPs underwent XY filtration using Hail[41] (v0.2.113) with benchmarking assessment performed using Hap.py[42] (v0.3.15). Genetic ancestry was called using germline SNPs and INDELs as inputs using ADMIXTURE[43] (v1.3.0) and PLINK[44,64] (2.00a4.5lm). Germline SVs were called using Delly2[45] (v1.2.6) and Manta[46] (v1.6.0). Mitochondrial SNVs were called using mitoCaller[3] (v1.0.0). Somatic SNVs were called using DeepSomatic[47] (v1.9.0), MuSE2[48] (v2.0.4), SomaticSniper[49] (v1.0.5.0), Strelka2[50] (v2.9.10) and Mutect2[39] (v4.5.0.0) followed by a consensus workflow to identify variants called by two or more callers using BCFtools[51] (v1.17) with quality control plots generated with BPG[52] (v7.1.0) and VennDiagram[53] (v1.7.4). Somatic SVs were called using SVision[54] (v1.4), Delly2[45] (v1.2.6) and Manta[46] (v1.6.0) and visualized with circlize[55] (v0.4.16). Somatic CNAs were called using CNV_FACETS[56] (v0.16.0) for the PCAWG sample and using Battenberg[57] (v2.2.9) for the TCGA sample with visualization generated using BPG[52] (v7.1.0). Taking the consensus set of somatic SNV calls and the CNA calls, subclonal reconstruction was performed using PyClone-VI[58] (v0.1.2), PhyloWGS[59] (v2205be1) and FastClone[60] (v1.0.9). Variant liftover was performed using BCFtools[51] (v1.20) and stability prediction using StableLift[24] (v1.0.0). Variant annotation was done using SnpEff[61] (v5.1d), Funcotator[39] (v4.2.4.1) and VEP[62] (v101.0) and ClinVar[37] (v20211016). Reconstructed phylogeny was visualized using CEV[63] (v2.0.0). Data validation was performed with PipeVal[29] (v5.1.0) and data processing was done using Nextflow[13] (v23.04.2).

Benchmarking analysis

Three variant-calling pipelines were assessed against metapipeline-DNA: nf-core/sarek,[34] Sentieon[35] and DRAGEN.[36] Qualitative assessment of pipeline features was performed with all pipelines with quantitative benchmarking focused against nf-core/sarek. Somatic variant calling assessment compared variant calls made by nf-core/sarek and metapipeline-DNA with a deep sequencing validation callset. For each pipeline, F1 scores were calculated based on variant calls made and resource usage was compared using the peak memory usage. Germline variant calling was assessed by comparison with an available benchmarking truth set of calls. For both pipelines, F1 scores and false discovery rates were calculated for the INDEL and SNP categories of germline variants.

QUANTIFICATION AND STATISTICAL ANALYSIS

Quantification of coverage was performed with two metrics. The percent of based exhibiting at least a given coverage depth were plotted from depths of 0× to 100×. Cohort-level coverage summaries were generated through plotting distributions of mean and median coverage values per sample. For assessment of consensus somatic SNV calls, VAFs across the variant callers were adjusted with a weighted average for each combination of 1, 2, 3 and 4 variant callers based on selection of variant sets called by the respective number of callers. Performance assessment of variant calling was done for each category of calls (somatic SNVs, germline INDELs, germline SNPs) using true positives (TP), false negatives (FN), false positives (FP), F1 score, false discovery rate (FDR) and sensitivity. Computational performance was quantified through comparison of pipeline runtime in real time and of peak memory usage.

Cell Reports Methods, Volume *6*

Supplemental information

Metapipeline-DNA: A comprehensive germline and somatic genomics Nextflow pipeline

Yash Patel, Chenghao Zhu, Takafumi N. Yamaguchi, Nicholas K. Wang, Nicholas Wiltsie, Nicole Zeltser, Alfredo E. Gonzalez, Helena K. Winata, Yu Pan, Mohammed Faizal Eeman Mootor, Timothy Sanders, Sorel T. Fitz-Gibbon, Cyriac Kandoth, Julie Livingstone, Lydia Y. Liu, Benjamin Carlin, Aaron Holmes, Jieun Oh, John Sahrmann, Shu Tao, Stefan Eng, Rupert Hugh-White, Kiarod Pashminehazar, Arpi Beshlikyan, Madison Jordan, Selina Wu, Mao Tian, Jaron Arbet, Beth Neilsen, Roni Haas, Yuan Zhe Bugh, Gina Kim, Joseph Salmingo, Wenshu Zhang, Aakarsh Anand, Edward Hwang, Anna Neiman-Golden, Philippa Steinberg, Wenyan Zhao, Prateek Anand, Raag Agrawal, Brandon L. Tsai, and Paul C. Boutros

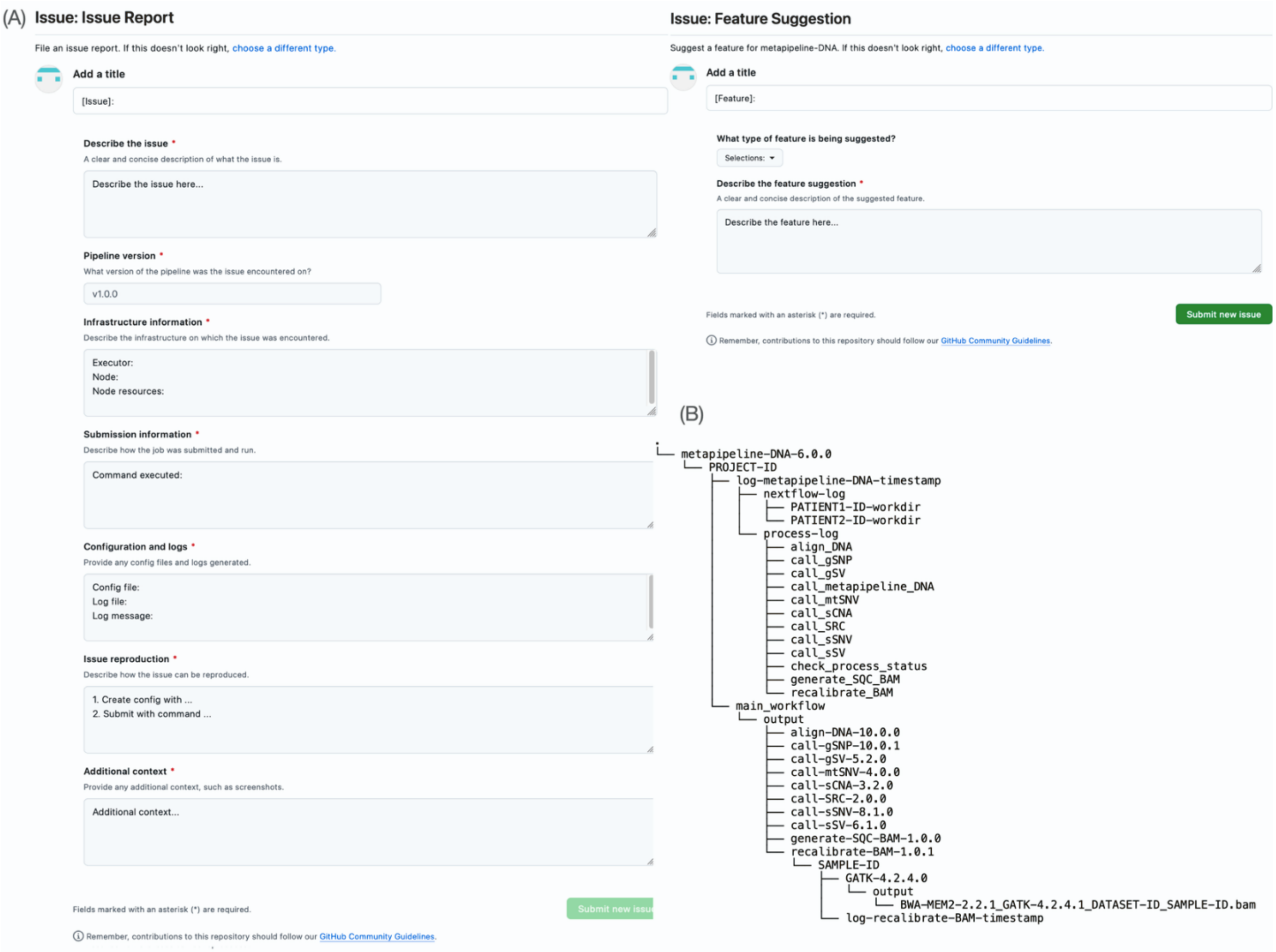

Supplementary Figure 1: **Reporting templates and output directory structure, related to Figure 1. (A)** Issue forms for submitting bug reports and feature suggestions, with structured input options to describe the bug/feature. **(B)** Outputs are organized under each pipeline with a sample/patient/project identifier followed by a directory for logs and a directory for each main tool used in the pipeline. Metapipeline-DNA outputs follow the same structure with individual pipelines' outputs organized recursively in metapipeline-DNA's output.

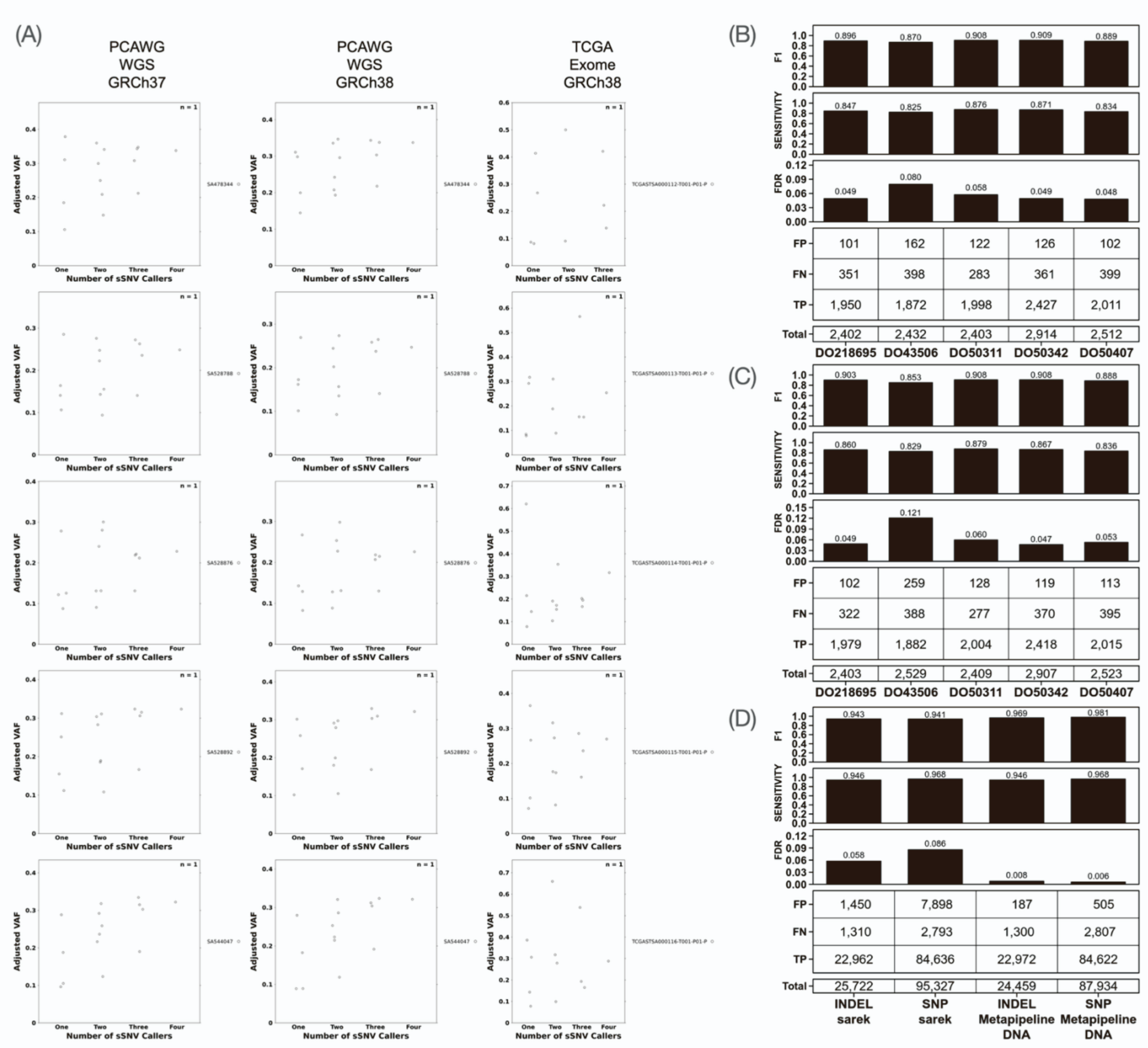

Supplementary Figure 2: VAF plots for all samples with variant calling comparison between sarek and metapipeline-DNA, related to Figures 1 and 2. (A) Variant allele frequencies based on consensus between callers for all samples processed. (**B)** TP, FN and FP variant calls comparing sarek Mutect2 SNV calls from the PCAWG-5 samples against a set of validation variant calls made from targeted deep-sequencing of the same samples. Numbers represent the number of variant calls. **(C)** TP, FN and FP variant calls comparing metapipeline-DNA Mutect2 SNV calls from the PCAWG-5 samples against a set of validation variant calls made from targeted deep-sequencing of the same samples. Numbers represent the number of variant calls. (**D)** TP, FN and FP variant calls comparing germline SNP and INDEL calls from sarek and metapipeline-DNA against the GIAB HG002 truth set. Numbers represent the number of variant calls.

Pipeline	Input Data	Sample Modes	Output Artefacts	Algorithms
Convert-BAM2FASTQ (https://github.com/uclahs-cds/pipeline-convert-BAM2FASTQ)	BAM/CRAM – Aligned reads in BAM or CRAM format	Single sample	FASTQ – Raw reads extracted per readgroup	SAMtools v1.15.1
Align-DNA (https://github.com/uclahs-cds/pipeline-align-DNA)	FASTQ – Paired raw reads with information about sequencing such as readgroup, library, sequencing center	Single sample	BAM – Aligned reads in BAM format	BWA-MEM2 v2.2.1<br>HISAT2 v2.2.1
Calculate-targeted-coverage (https://github.com/uclahs-cds/pipeline-calculate-targeted-coverage)	BAM – Aligned reads in BAM format<br>Target region BED – Genomic sites targeted for sequencing	Single sample	Expanded regions<br>Per-base depth in target regions and dbSNP sites<br>Hybrid-selection metrics	SAMtools v1.16.1<br>BEDtools v2.29.2
Recalibrate-BAM (https://github.com/uclahs-cds/pipeline-recalibrate-BAM)	BAM – Aligned reads in BAM format<br>*Target regions – Genomic sites targeted for sequencing/analysis*	Single sample<br>Normal-tumour paired samples<br>Multi-normal and/or multi-tumour samples	INDEL realigned and base-quality score recalibrated BAM	GATK v3.7.0, v4.2.4.1
Generate-SQC-BAM (https://github.com/uclahs-cds/pipeline-generate-SQC-BAM)	BAM – Aligned reads in BAM format (typically including INDEL realignment and BQSR)	Single sample	BAM statistics – Statistics related to alignment, reads, quality, duplication<br>Coverage metrics – Statistics and plots of coverage across the genome	SAMtools v1.18<br>Picard v3.1.0<br>Qualimap v2.3
Call-gSNP (https://github.com/uclahs-cds/pipeline-call-gSNP)	BAM - Aligned reads in BAM format (typically including INDEL realignment and BQSR)<br>*Target regions - Genomic sites targeted for sequencing/analysis*	Single sample<br>Normal-tumour paired samples<br>Multi-normal and/or multi-tumour samples	Per-sample GVCF – Genomic VCF generated per sample<br>Germline SNP VCF – Recalibrated and filtered germline SNP calls for set of given samples	GATK v4.2.4.1<br>DeepVariant v1.9.0
Call-mtSNV (https://github.com/uclahs-cds/pipeline-call-mtSNV)	BAM/CRAM – Aligned reads in BAM or CRAM format (typically including INDEL realignment and BQSR)	Single sample<br>Norma-tumour paired samples	Mitochondrial SNV VCF	MToolBox v1.2.1-b52269e<br>mitoCaller v1.0.0
Call-gSV (https://github.com/uclahs-cds/pipeline-call-gSV)	BAM – Aligned reads in BAM format (typically including INDEL realignment and BQSR); single normal BAM	Single sample	Germline SV BCF – Variant calls made by DELLY in BCF format<br>Germline SV BCF – Variant calls made by Manta in VCF format	DELLY v1.2.6<br>Manta v1.6.0
Call-sSV https://github.com/uclahs-cds/pipeline-call-sSV)	BAM – Aligned reads in BAM format	Normal-tumour paired samples	Somatic SV BCF – Variant calls made	DELLY v1.2.6<br>Manta v1.6.0<br>SVision v1.4

	(typically including INDEL realignment and BQSR)		by DELLY in BCF format<br>Somatic SV VCF – Variant calls made by Manta in VCF format	
Call-sSNV<br>https://github.com/uclahs-cds/pipeline-call-sSNV)	BAM – Aligned reads in BAM format (typically including INDEL realignment and BQSR)<br>*Somatic SNV calls – Variant calls provided in VCF format to run the consensus call workflow*<br>*Panel of normal – PON used with Mutect2 to improve variant calls*	Single tumour sample<br>Normal-tumour paired samples<br>Multiple tumour samples	Somatic SNV VCFs – Variant calls made by each of the algorithms, VCFs separated per algorithm and per variant type (SNV, MNV, INDEL) when applicable	Mutect2 v4.5.0.0<br>Strelka2 v2.9.10<br>SomaticSniper v1.0.5.0<br>MuSE v2.0.4<br>DeepSomatic v1.9.0<br>BCFtools-Intersect v1.17
Call-sCNA<br>(https://github.com/uclahs-cds/pipeline-call-sCNA)	BAM – Aligned reads in BAM format (typically including INDEL realignment and BQSR)	Normal-tumour paired samples	Somatic CNA TSV – Aberrations called by Battenberg in TSV format<br>Somatic CNA VCF – Aberrations called by FACETS in VCF format	Battenberg v2.2.9<br>FACETS v0.16.0
Call-SRC<br>(https://github.com/uclahs-cds/pipeline-call-SRC)	SNV calls – Generated by any of the algorithms from call-sSNV<br>CNA calls – Generated by any of the algorithms from call-sCNA and HATCHet	Single tumour sample<br>Multiple tumour samples	SNV clustering – Result of clustering of SNVs by clustering algorithms<br>Reconstructed phylogeny	PyClone v0.13.1<br>PyClone-VI v0.1.2<br>PhyloWGS v2205be1<br>DPClust v75f5d7e<br>FastClone v1.0.9<br>CliP v1.3<br>CONIPHER v2.2.0
StableLift<br>(https://github.com/uclahs-cds/pipeline-StableLift)	Variant calls – Generated by any of the following algorithms: HaplotypeCaller, Mutect2, Strelka2, SomaticSniper, MuSE2, DELLY2	Single sample	Lifted variant calls – Variant calls lifted over into the target reference genome<br>Variant stability score – Predicted score of variant stability across reference genome builds	BCFtools v1.20<br>StableLift v1.0.0
Call-GeneticAncestry<br>(https://github.com/uclahs-cds/pipeline-call-GeneticAncestry)	Germline variant calls – Generated by any germline variant caller	Cohort of samples	Predicted genetic ancestry	ADMIXTURE v1.3.0<br>PLINK2 v2.00a4.5lm
Annotate-VCF<br>(https://github.com/uclahs-cds/pipeline-annotate-VCF)	Variant calls – Generated by any caller in VCF format	Single sample	Annotated variant calls – Variant calls annotated with the selected databases	SnpEff v5.1d<br>Funcotator v4.2.4.1<br>VEP v101.0
Calculate-mtDNA-CopyNumber<br>(https://github.com/uclahs-	Genomic coverage – Coverage information per-contig to be used	Single sample	Calculated mitochondrial DNA copy number	

cds/pipeline-calculate-mtDNA-CopyNumber)	in calculating mitochondrial DNA copy number			

Supplementary Table 1: Detailed pipeline inputs, outputs, and tools, related to Table 1. Detailed description of inputs, outputs, run modes, and tools encompassed in metapipeline-DNA. Inputs that are *italicized* are optional and inputs separated by "/" represent a list of choices from which one must be chosen.

**Pipeline**	**PCAWG WGS GRCh37 (wall-clock time in hours)**	**PCAWG WGS GRCh37 (Peak RAM in GB)**	**PCAWG WGS GRCh38 (wall-clock time in hours)**	**PCAWG WGS GRCh38 (Peak RAM in GB)**	**TCGA WXS GRCh38 (wall-clock time in hours)**	**TCGA WXS GRCh38 (Peak RAM in GB)**
Align-DNA (normal)	4.92 ± 1.68	38.12 ± 1.36	5.56 ± 2.04	50.82 ± 1.73	0.40 ± 0.12	24.82 ± 0.69
Align-DNA (tumour)	7.06 ± 0.69	38.48 ± 1.98	8.20 ± 0.86	51.42 ± 5.07	0.39 ± 0.09	24.96 ± 0.62
Recalibrate-BAM	31.01 ± 4.64	21.20 ± 0.44	31.31 ± 3.08	20.62 ± 0.06	2.10 ± 0.44	2.84 ± 0.07
Generate-SQC-BAM	3.80 ± 2.17	0.41 ± 0.66	4.96 ± 0.67	1.53 ± 0.08	0.34 ± 0.07	0.92 ± 0.009
Call-gSNP	13.14 ± 1.16	5.36 ± 0.07	7.27 ± 3.41	5.42 ± 0.06	0.41 ± 0.11	5.06 ± 0.07
Call-mtSNV	3.43 ± 0.17	10.56 ± 4.06	3.23 ± 0.42	10.58 ± 4.01	0.096 ± 0.024	6.46 ± 0.11
Call-sSNV	12.04 ± 2.92	48.54 ± 2.30	10.52 ± 1.83	43.7 ± 6.98	0.43 ± 0.07	29.32 ± 3.82
Call-sSV	12.85 ± 6.60	9.47 ± 6.55	18.89 ± 3.01	13.14 ± 0.29	0.44 ± 0.11	8.60 ± 0.91
Call-gSV	7.33 ± 2.09	7.24 ± 1.90	8.05 ± 2.83	7.48 ± 1.80	0.34 ± 0.08	2.10 ± 0.09
Call-sCNA	3.43 ± 0.17	46.0 ± 5.67	3.23 ± 0.42	45.14 ± 5.89	2.72 ± 0.05	19.26 ± 0.07
Call-SRC	2.39 ± 0.96	0.41 ± 0.09	2.58 ± 1.01	0.41 ± 0.09	0.014 ± 0.002	0.27 ± 0.04
**TOTAL**	**83.36 ± 12.99**	**-**	**81.76 ± 14.23**	**-**	**6.05 ± 0.80**	**-**

Supplementary Table 2: Runtime and peak physical memory usage of pipelines per sample with 95% confidence intervals, related to Table 1. The total runtime is less than the sum of the individual pipelines' runtimes due to parallelization of variant calling pipelines.
